# Agrobiological Interactions of Essential Oils of Two Menthol Mints: *Mentha piperita* and *Mentha arvensis*

**DOI:** 10.3390/molecules25010059

**Published:** 2019-12-23

**Authors:** Danuta Kalemba, Agnieszka Synowiec

**Affiliations:** 1 Institute of Natural Products and Cosmetics, Lodz University of Technology, 90-924 Łódź, Poland; danuta.kalemba@p.lodz.pl; 2 LeStudium Institute for Advanced Studies, 45000 Orléans, France; 3 Department of Agroecology and Crop Production, University of Agriculture in Kraków, 31-120 Kraków, Poland

**Keywords:** agriculture, botanical pesticides, chemical composition

## Abstract

This review article discusses the active constituents and potential of two menthol mint oils, *Mentha piperita* (MPEO) and *Mentha arvensis* (MAEO), as natural sources for botanical pesticides. The biological activities of these menthol mint oils, which can be useful in agriculture, have been broadly researched, especially toward phytotoxic microorganisms. To a lesser extent, the insecticidal and herbicidal activities of mint EOs have also been studied. It is apparent that the prospect of using menthol mint oils in agriculture is increasing in popularity. A number of investigations showed that the in vitro efficacy of MPEO and MAEO, as well as that of their main constituent, menthol, is pronounced. The results of in vitro research are useful for choosing EOs for further investigations. However, it is clear that in situ experiments are crucial and should be more extensively developed. At the same time, known techniques are to be applied to this area and new methods should be worked out, aiming at the improvement of EOs’ pesticidal efficacy and cost-effectiveness, for future implementation in agricultural pest control.

## 1. Introduction

The genus mint (*Mentha*) belongs to the Lamiaceae family and includes 42 species, 15 hybrids, and hundreds of subspecies, varieties, and cultivars [1], which potentially crossbreed when in proximity. Different mints are known for a reasonably high content of essential oils (EO), which are deposited in the glandular trichomes, mostly located on the adaxial surface of their leaves [2]. There are two well-known, so-called menthol mints in cultivation: *Mentha x piperita* L. (Hudson): peppermint—MP, and *Mentha arvensis* L., (syn. *M. canadensis* L., Japanese mint): cornmint—MA [3].

MP originates from the Mediterranean region and is a natural hybrid between *M. viridis* (*M. longifolia* x *M. rotundifolia*) and *M. aquatica* [4]. It has higher yields in the temperate climate regimes of higher precipitation levels. A widely cultivated botanical form of MP is *Mentha x piperita* L. var. officinalis Sole f. rubescens (Camus), called black or English MP, which has violet stems and leaves [5,6]. MP can be grown as a sole crop or intercropped with other species [7,8,9]. A recent study showed that the inclusion of MP in crop rotation can negatively affect a succeeding maize, which may result from the allelopathic interactions of MP [10], possibly due to changes in the quantitative and qualitative profiles of EO during its decomposition in the soil [11].

MA originates from the temperate climates of Europe and western and central Asia. It has higher yields under the subtropical conditions of Asia [12,13]. MA is usually included in the crop rotation with different crop species as it reacts well to intercropping and green manuring [14].

The popularity of MP and MA cultivation results from a wide application of both herbs and essential oils. Due to the biological quality of the raw material obtained from plantations, and its use for medicinal purposes, ecological cultivations of both menthol mints are recommended [15,16,17,18,19,20].

The menthol mints contain many biologically active compounds, with EOs being a significant part of them. The biological interactions of the menthol mints with the other components of agrobiocenoses, i.e., weeds or insect pests, have been observed for a long time. Recently, the investigation of the menthol mints EOs as natural (aka botanical) pesticides is being carried out. Peppermint essential oil is already exempt from the Federal Insecticide, Fungicide, and Rodenticide Act (FIFRA) as a pesticide formulation, alone or in combination with other ingredients [21].

Nowadays in agriculture there is a growing interest in botanical pesticides with the active ingredient composed of natural compounds, among them EOs [22,23]. This is due to two major factors. Firstly, the misuse of synthetic pesticides has resulted in the rise of a number of pesticide-resistant organisms, which are also posing a significant threat to the diversity of ecosystems [24,25]. Indeed, the contamination of environment and biological systems with overused synthetic products led to the establishment of the REACH ordinance (1907/2006/WE) in the European Union. As a result, the most hazardous pesticides are being withdrawn for the European market and replaced with suitable natural alternatives. Secondly, increasing consumer awareness of the negative impact of synthetic pesticides on human health boosts the development of natural products with different biological activities. For all of the above reasons, EOs are perceived as good alternatives to synthetic pesticides.

Based on the recent scientific literature, a critical review of the chemical composition of peppermint oil (*Mentha piperita*, MPEO) and cornmint oil (*Mentha arvensis*, MAEO), and their potential for being incorporated in future botanical pesticides, is presented below.

## 2. Content and Chemical Composition of Peppermint Oil and Cornmint Oil

Essential oils are multicomponent mixtures of secondary plant volatiles produced by steam- or hydrodistillation of different plant parts, with the exception of citrus peel oils, which are produced by expression. The main constituents of essential oils belong to the mono- and sesquiterpenes, which are classified into hydrocarbons, alcohols, aldehydes, ketones, esters, and ethers. Essential oils are limpid, oily liquids that dissolve well in ethanol, unpolar organic solvents, and lipids, and are insoluble in water.

MPEO and MAEO containing the same major constituents, namely menthol and menthone, are among the most produced and marketed essential oils all over the world. The main producers of MPEO are India, the USA, and China, and of MAEO China, India, Brazil, and Japan [26,27]. The oils are obtained by hydrodistillation of the fresh or partly dried flowering herb with a yield of 0.3–0.7%. In both EOs about 300 constituents were identified. The main constituents of MPEO are menthol (20–60%), menthone (5–35%), menthyl acetate (1–20%), and menthofuran (0.1–15%). MAEO is dominated by menthol (above 60%) and menthone (4–18%). Menthol is separated from this oil by crystallization and the remaining oil has an appearance and odor resembling MPEO. The dementholized MAEO is used as a cheap alternative to MPEO, but it is easily recognized organoleptically because of its harsh flavor.

**Figure 1:**
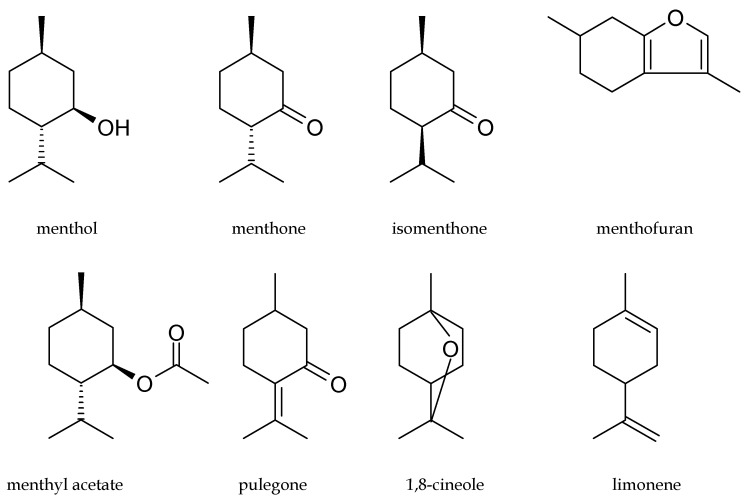
Structures of the main components of menthol mint essential oils.

Both menthol-rich mint oils have monographs in the *European Pharmacopoeia* 5 (*EP* 5) [28] as Peppermint oil and Mint oil partly dementholized, respectively. EP 5 defines mint oils as colorless, pale yellow, or pale greenish-yellow liquids with a characteristic odor. EP 5 establishes the limits of 10 key components in peppermint oil determined by GC analysis: menthol (30.0–55.0%), menthone (14.0–32.0%), isomenthone (1.5–10.0%), menthyl acetate (2.8–10.0%), menthofuran (1.0–9.0%), 1,8-cineole (3.5–4.0%), limonene (1.0–5.0%), isopulegol (max. 0.2%), pulegone (max. 4.0%), and carvone (max. 1.0%). The limits of these compounds in dementholized cornmint oil are similar: menthol (30–50%), menthone (17–35%), isomenthone (5.0–13.0%), menthyl acetate (1.5–7.0%), 1,8-cineole (max. 1.5%), limonene (1.5–7.0%), isopulegol (1–3%), pulegone (max. 2.0%), and carvone (max. 2.0%). The structures of the main constituents of menthol mint oils are presented in Figure 1.

The yield as well as the qualitative and quantitative composition of MPEO differs in relation to cultivar, geographic origin, and condition of cultivation (temperature, water, fertilizers), and strongly depend on the time of harvest. At the beginning of ontogenesis, the herb contains menthone (40–55%) as a main compound and lower amounts of menthol (20%). During shoot growth, the menthol content starts to increase, reaching more than 40%, while the menthone content decreases. At the flowering stage, the content of two other constituents, namely menthofuran and pulegone, which adversely influence peppermint oil quality, increases and diminishes after flowering, while the content of menthol and menthyl acetate increases to levels higher than 50% and 7%, respectively [29]. The conditions of growth of MP in cultivation may additionally affect the quality of MPEO. For example, organic fertilizers promote the production of EO of a higher amount of menthol and a decreasing amount of menthofuran and pulegone [30,31]. The amount of menthol can also be increased as a result of microbial activity in the MP’s rhizosphere. For example, an increase in the number of native rhizospheric strains of bacteria *Pseudomonas putida* and their microbial volatile organic compounds stimulated MP’s shoot growth and reduced the content of menthofuran in the EO, with a simultaneous induction of menthol production [32]. A similar effect brought about the arbuscular mycorrhizal inoculation of soil with *Funneliformis mosseae*, which, when applied alongside foliar-sprayed natural humic substances, promoted the biochemical activity of MP plants [33].

The rhizosphere of MA is also rich in several different strains of mycorrhizal fungi, which positively influence the production of MAEO, specifically the menthol content. Interestingly, the highest production of menthol was achieved when MA plants were inoculated with *Trichoderma viride* [34].

Menthol is a monoterpene alcohol with three chiral carbon atoms and occurs in eight stereoisomes. In both mint oils, (−)-(1*R,*3*S,*4*S*)-menthol, called menthol, is dominant. Three dextrorotatory menthol isomers, (+)-(1*S,*3*R,*4*S*)-isomenthol, (+)-(1*R,*3*R,*4*R*)-neomenthol, and (+)-(1*R,*3*R,*4*S*)-neoisomenthol, are present in the oils in smaller amounts. Out of four stereoisomers of appropriate ketone, (−)-(1*R,*4*S*)-menthone dominated over (+)-(1*R,*4*R*)-isomenthone.

In a recent review on the genus *Mentha*, previous literature data on MPEO and MAEO composition were reported [1]. Only five MPEOs met the requirements of *EP* 5 in respect of the main constituents’ percentages. Five oils have components from the *EP* 5 list as the main constituents, but in different proportions. The other oils were composed of totally different compounds. In two of them, carvone and limonene were the main constituents, as in *M. spicata* oil, while in three oils linalool and linalyl acetate dominated. Similarly, among the three MAEOs there were oils dominated by menthol/isomenthone, menthol/pulegone, or linalool/linalyl acetate [1].

## 3. Biological Activity and Application of Peppermint Oil and Cornmint Oil

MPEO is the most important of the mint oils because of its exceptional properties [26,35,36]. It is also the most extensively used oil in therapy, both internally and externally, being recommended for the treatment of acute and chronic gastritis and enteritis, in disorders of the respiratory tract, and for inflammation of the oral mucosa [26]. The biological activity of menthol mint oils is due to the content of their main constituent menthol, which is used as an individual phytochemical in the treatment of respiratory pathologies. Both MPEO and menthol are ingredients in numerous medications.

MPEO possesses a fresh, minty flavor and cooling effect. Due to these properties and its antimicrobial activity, it is also widely used in chewing gums, toothpastes, and mouthwashes, and as a fragrance in perfumes, soaps, and air refreshers, where it is often replaced by a cheaper, dementholized MAEO.

## 4. Antifungal and Antibacterial Activity of Peppermint Oil and Cornmint Oil against Phytopathogens

The wide spectrum of therapeutic properties of peppermint oil includes antibacterial and antifungal activities. Due to these activities, MPEO and MAEO are also used for controlling microorganisms in other areas. In the last few decades, the use of EOs in agriculture, as agents protecting crops from bacterial and fungal diseases, has been extensively researched.

Two basic techniques are used for the in vitro assessment of antibacterial and antifungal activities of EOs. In the agar diffusion method, agar broth is inoculated with microorganisms and EO or EO solution is placed on a paper disc or in a well. After incubation, the diameter of the inhibition zone is measured. In the serial dilution agar or liquid broth method, EO is added to the broth, which is inoculated with microorganisms. In fungi this method, called a poisoned food technique, is used for the assessment of mycelial growth inhibition at specified EO concentrations. In both variants, the activity of EOs can be assessed in a vapor phase. The results are presented in terms of the growth inhibition as a percentage ratio to the control or as the minimal inhibitory concentration (MIC) restraining microorganism growth. Sometimes the bactericidal (MBC) or fungicidal (MFC) concentration is also assessed. A negative control without EO and positive control with standard antibiotics for bacteria and fungicide for fungi should be included in the experiment. It should be mentioned that the results obtained in different laboratories are hardly comparable because of a high number of factors influencing the final result. Among them, the origin and susceptibility of microorganisms, i.e., environmental fungi and bacteria are more resistant than collection strains, and conditions of assessment, i.e., method, solvent, MIC definition, and different units of EO concentration, are the most important [37,38]. To a broad extent, MIC values can be compared between laboratories. On the contrary, inhibition zones measured by disc diffusion method are incomparable because of the varying EO amounts used.

The results of MPEO and MAEO antimicrobial activity investigated by in vitro methods against phytopathogenic fungi and bacteria are presented in Table 1, with an emphasis on the results obtained by the dilution method. In the majority of studies, several EOs were assessed in one study. For comparison purposes, the data for the most active EO are also presented. Different units used for the EO concentration (mg/mL, μL/mL, μL/L, ppm, etc.) were converted to the same unit, μg/mL, on the assumption that EO density amounts to 1 g/mL. In fact, it is ca. 0.9 g/mL [28].

The antimicrobial effectiveness of MPEO was assessed more often, and the spectrum of tested plant-pathogenic microorganisms was broader than that of MAEO. The research applied to antifungal activity predominated over bacterial activity.

The antifungal and antibacterial activity of MPEO and MAEO, expressed as the MIC value, was, in the majority of studies presented in Table 1, in the range of 0.25–3 μL/mL 250–3000 μg/mL). However, in some cases the MIC was about 10 times lower, at 44–149 μg/mL [39,40], or even hundreds of times lower, 0.5–10 μg/mL [41]. In the latter research, the MIC values of MPEO were lower for five fungi, the same for two, and for others higher than that of synthetic fungicide amphotericin [41]. MPEO and MAEO were additionally proven to reveal antimicrobial activity in numerous disc diffusion tests.

In research in which series of EOs were investigated, menthol mint oils usually belonged to the group of highly or moderately effective oils. Among 32 essential oils, only MPEO and basil oils were effective in a disc diffusion assay at 20 and 50 μL, respectively, against the *Acidovorax citrulli* bacterium that caused fruit blotch in watermelon [42]. Similarly, MAEO was the most effective against nine fungi out of 18 EOs. The oil at 0.1 mg/mL (100 μg/mL) totally inhibited the growth of four fungi and showed 72–100% inhibition of five others. The highly sensitive fungi were *Aspergillus flavus*, *Helmithosporium oryzae*, and *Sclerotium rolfsii*, with MIC 0.1 mg/mL (100 μg/mL). MPEO was more effective against two toxigenic *A. flavus* strains than four synthetic fungicides [40]. In other research, MAEO was the only one out of 18 EOs that totally inhibited 11 fungal strains at 1000 μg/L (1 μg/mL), with an MIC at 400 μg/L (0.4 μg/mL) toward nine strains being more efficient against *A. flavus* than 10 synthetic fungicides that had the MICs in a range 500‒2000 μg/L (0.5‒2 μg/mL) [43]. From 105 samples of essential oils representing 53 plant species, MPEOs (20 samples) were among the 18 species exhibiting the highest antifungal activity. When introduced at 1 and 10 μL/mL (1000 and 10,000 μg/mL) to the broth, MPEOs caused a 70–98% reduction of *Aspergillus niger* and *A. ochraceus* and a 47–85% reduction of *Fusarium culmorum* mycelial growth [44]. In an activity assessment of eight EOs against three plant-pathogenic fungi, *Phytophthora cinnamomi, Pyrenochaeta lycoprsici,* and *Verticillium dahliae*, only oregano and thyme oil were more active than MPEO, while the other five oils showed lower activity [45]. Among the 10 EOs assessed against mushroom pathogens, the fungi *Trichoderma harzianum* and *Verticillium fungicola* and the bacterium *Pseudomonas tolaasii*, only the thyme and oregano oils (MIC 1.5–2.0 μL/mL = 1500–2000 μg/mL) were more effective than MPEO (MIC 3–4 μL/L = 3000–4000 μg/mL), which showed better activity than bifonazole against fungi and almost the same activity as the streptomycin and penicillin mixtures against *P. tolasii* [46]. Similarly, among 18 EOs only three were more efficient than MPEO against five fungal strains isolated from fruits [47]. MPEO was in the group of moderate activity among the 45 EOs researched against three fungi and eight bacteria strains by the disc diffusion method [48]. On the other hand, MPEO appeared the least active out of four EOs against *Fusarium moniliforme* [49] and showed poor efficacy against two plant-pathogenic bacteria, *Agrobacterium tumefaciens* and *Erwinia carotovora* [50].

In spite of quite good antifungal activity against *Lecanicillium fungicola* var. *fungicola*, a fungus that causes dry bubble disease in the mushroom *Agaricus bisporus*, MPEO was not suitable for mushroom protection because of similar activity against fungi, MIC 750–1000 μL/L (750–1000 μg/mL). Among 11 EOs, activity toward mushrooms and pest mycelial growth were assessed in a broth dilution assay, savory (carvacrol 38%) and thyme (carvacrol 46.1%, thymol 30.4%) oils showed the best selectivity index, i.e., were more inhibitive to the growth of the pathogen (MIC 200–250 μg/mL) in comparison to the mushroom (MIC 400 μg/mL) [51].

Fungal toxins are common contaminants in grains, fruits, and vegetables during storage. EOs play a role not only in the reduction of fungal growth, but also in the inhibition of toxin production. MAEO at 1000 ppm completely inhibited the fungal growth of *A. ochraceus* and ochratoxin A production for up to 21 days [52]. Hua et al. [53], in research on five EOs and five compounds against *A. ochraceus* growth and ochratoxin production, showed that cinnamon oil and cinnamaldehyde were the most effective. They did not investigate ochratoxin production in the presence of MPEO. However, they proved that MPEO inhibited fungal growth at 1500 μL/L and the decrease in ochratoxin production by other oils was proportional to the decrease in fungal biomass and correlated with ergosterol inhibition. In other research, MAEO completely inhibited aflatoxin B1 production by the toxigenic strain of *A. flavus* at 0.05 mg/mL, while the radial mycelial growth of this strain was stopped by 0.1 mg/mL [40].

Four MPEOs of different origin and small differences in quantitative composition (main components in accordance with *EP* 5 demands) showed weak antifungal activity in an agar diffusion test. On the other side, the oils strongly inhibited plant-pathogenic bacteria in a dilution test. Pathovars of *Pseudomonas syringae* and *Xanthomonas campestris* differed in terms of their susceptibility to the oil. For some bacterial strains, correlations were found between the oil activity and menthol and menthone percentages [54].

Hussain et al. [39] investigated the content, composition, and antimicrobial activity of four mint species EOs in two harvesting seasons, summer and winter. The authors observed variation in all aspects. However, they stated that, along with the changes in EOs composition depending on the planting time and mineral fertilization, the oils showed a different degree of inhibition: the oils from crops planted and fertilized in the spring were more active against some bacteria. The authors concluded that MAEO exhibited the highest antifungal and antibacterial activity in both tested methods (disc diffusion and broth microdilution), while MPEO, *M. longifolia*, and *M. spicata* oils revealed a similar efficacy [39].

The antifungal and antibacterial activity of MAEO (78.9% menthol) was assessed by the disc diffusion method and compared with the activity of fractions obtained from this oil: dementholized EO (DMAEO, 28.1% menthol), monoterpenes (mainly α- and β-pinene, limonene, and myrcene), menthol, menthone, and isomenthone. At a dose of 5 μL per disc, MAEO and monoterpene fraction showed the highest activity against *A. fumigatus* and *A. niger* (IZ 12–15 mm), followed by DMAEO (IZ 7–11 mm). Similarly, the highest activity against 12 bacterial strains was observed for monoterpenes, MAEO, and DMAEO [55].

In general, the most antimicrobial EOs are oregano, thyme, and savory oils. In the presented research these EOs were shown to be more effective than both menthol mint oils [39,45,46,47,51]. The activity of any EO is strictly connected with its composition. Thyme, oregano, and savory oils contained, as their main constituents, monoterpene phenols, carvacrol, and thymol, which showed higher activity against fungi [56,57] and bacteria strains [50] than menthol. However, there are exceptions to this rule. In nine foodborne fungal pathogens, menthol was more effective to *Penicillium citrinum* than both phenols and similarly effective to *A. ochraceus* (MIC 100 μg/L, MFC 125) [58]. Among 10 monoterpenes, the efficacy of menthol against three fungal pathogens of mushroom was the same as that of thymol and carvacrol, and better than that of other compounds [46].

The antimicrobial activity of MPEO and MAEO is definitively attributed to the presence of menthol, which in all studies was shown to be more effective than menthone. When 22 compounds were tested against *Botrytis cinerea* and *Monilinia fructicola* conidial germination and mycelial growth in broth culture, thymol and carvacrol showed total inhibition at 100 μg/mL, while menthol at 250 μg/mL showed 96% and 97% inhibition and menthone 45% and 8% inhibition of conidial germination of *B. cinerea and M. fructicola*, respectively. At 100 μg/mL, menthol was effective against *M. fructicola* (95% inhibition) and less effective against the mycelial growth of *B. cinerea* (47% inhibition) [57]. Menthol belonged to a group of the eight most active compounds in the set of 21 EO constituents assessed by the disc diffusion method toward 10 Gram+ and 20 Gram− bacterial strains. The most susceptible were *Aerococcus viridans*, *Clavibacter michiganense*, *Kocuria varians*, two of seven *P. syringae* pathovars, two of four *Erwinia* spp., three *Xanthomonas* taxa, *Neisseria subflava*, and *Agrobacterium tumefaciens*. None of the compounds was effective against all strains. Menthol inhibited the growth of 16 strains but menthone of two strains only [59]. The antimicrobial activity of the main mint oil constituents against seven plant-pathogenic fungi strains was compared with the activity of the standard drug fuconazole in a microdilution assessment The menthol activity (MIC 30.8–107.7 μg/mL) was similar to that of fluconazole (MIC 10.4–100 μg/mL). Menthone, carvone, and piperitenone oxide showed lower activity [39]. According to these reports, it seems that the higher antimicrobial effectiveness of MAEO as compared to MPEO could be attributed to a higher content of menthol, which is more active than menthone.

Chirality is an important aspect of EO compounds because enantiomers may possess different biological activity. According to recent research, in the case of antimicrobial activity, the essential oil constituents’ chirality seems to be insignificant. Only a few studies have been performed on that topic. No differences were observed in the activity against three bacteria strains between (−)- and (+)-menthol. However, (+)-menthol was significantly more active than its enantiomer against *Aspergillus brasiliensis* [72].

## 5. Peppermint Oil and Cornmint Oil for Postharvest Decay Control

Fruit and vegetables are important components of the human diet and are mostly marketed as fresh. Due to their high moisture content, they are highly susceptible to attack by different pathogenic bacteria and fungi, causing diseases that lead to high produce loss during harvest, transport, and storage. Similarly, microbial contamination is an important factor in the safety of cereals. The appropriate treatments for controlling postharvest decay of fruits and vegetables may diminish their losses and improve their quality, safety, and shelf life. It is commonly accepted that EOs are more effective in in vitro models than in in situ models, e.g., in food or on the field. The interest in natural agents for decay control in fruit, vegetables, and grains needs not only in vitro methods for searching for appropriate active substances. Hence, more and more often, in vitro studies are supported by experiments performed in a real environment, usually referred to as in vivo methods; however, in our opinion, these methods should be called in situ.

In a review on the use of EOs for postharvest decay control, Antunes and Cavaco [73] presented the main diseases of fruits and vegetables and the essential oils used against them. They mentioned menthol but not mint oils. In this section the results for the efficacy of both menthol mint EOs as well as their main constituents in a real environment are summarized.

The most common fungal plant pathogens are species of *Aspergillus*, *Alternaria*, *Fusarium*, *Penicillium*, *Sclerotinia*, *Rhizopus*, and *Botrytis cinerea.* Bacterial decay agents include species of *Erwinia*, *Pseudomonas*, *Xanthomonas*, and *Bacillus*. These plant-pathogenic microorganisms and a broad spectrum of other ones were included in numerous assessments of menthol mint oils’ efficacy for pre- and postharvest decay control. The majority of research applied to fruits. Fruits not treated or inoculated by pests were either immersed in an active agent solution or exposed to EO vapors. In some cases, in situ research was preceded by in vitro research. The details of the research and the results are presented in Table 2.

Lopez et al. [74,75] investigated the efficacy of 11 essential oils on the postharvest control of rot caused by fungi *B. cinerea* and *Penicillium expansum* on four cultivars of apples [74] and by *B. cinerea* and *Monilinia laxa* on different cultivars of apricots, nectarines, and plums [75]. In both studies, wounded fruits were inoculated with a spore suspension and, after drying, 10 μL of 1% (10,000 μg/mL) and 10% (100,000 μg/mL) EO emulsion was dropped into each wound. Ten microliters of the tebuconazole suspension (125 μg/mL) was used as a chemical control. The efficacy against gray mold strongly depended on EOs, the fruit cultivar, and the storage time. The most effective thyme, oregano, and savory EOs controlled fungal growth in apples, apricots, nectarines, and plums at 1%. MPEO and MAEO revealed good efficacy at 10%, similar to or better than other EOs. However, the EOs’ efficacy was statistically lower than tebuconazole and, at this concentration, the EOs were also phytotoxic to fruit.

Ziedan and Farrag [76] isolated the three most common fungi that caused rot syndromes of peaches at local markets in Egypt, *Rhizopus stolonifer*, (56.5%), *M. fructicola* (17.1%), and *A. niger* (7.5%), and tested the pathogenicity of MPEO, sweet basil oil, and the main components of the oils individually and in mixtures. Vapors of both oils in an in vitro assay completely inhibited *R. stolonifer* and *M. fructicola* at doses of 30 μL/400 cm^3^ air and 20 μL/400 cm^3^ air, respectively. Vapors of oils and blends of the major individual constituents’ ratio similar to their concentrations in the original oil (mentone and menthol) inhibited the growth of the fungal pathogens. Mixing two components for each oil was found to enhance the antifungal properties (in a lower dose), indicating a synergistic effect. The optimal oil dose to reduce decay and maintain fruit quality after prolonged storage was 3 mL/box.

MAEO was a potent fumigant for the management of the biodeterioration of stored oranges [77]. At a concentration of 1500 ppm in vapor, the oil prevented the decay of oranges inoculated by fungi that caused blue mold rot disease in fruit and enhanced the storage life from three to 10 days. In other research [78], the fungitoxic activity of MAEO was much higher. The oil, at a concentration of 100 ppm, controlled fungal growth in oranges and limes inoculated by *P. italicum* and enhanced the storage life of oranges to nine days and of limes to 11 days, compared to three days in the control. MAEO was the most efficient out of the 20 EOs tested by the agar dilution method (MIC 100 ppm = 100 μg/mL). The MIC of the four synthetic fungicides was higher than the EOs tested (1000–5000 ppm = 1000–5000 μg/mL).

MPEO and rosemary oil, alone or in combination, effectively controlled the incidence of gray mold in table grapes exposed for 24 h to EO vapors both in atmospheric pressure and in hypobaric treatment (50 kPa) [79]. The grapes were then stored at room temperature for five or nine days, and at 4 °C for seven days, followed by three days’ shelf life at 2 °C. EO smell and taste were not perceived on grapes by a tasting panel after 48 h.

The effectiveness of EOs may be different in in vitro and in vivo models. Four essential oils (*M. piperita*, *Lavandula angustifolia*, *Cuminum cyminum*, and *Foeniculum vulgare*) were tested on three fungi that cause major losses of strawberries during storage, *A. niger, B. cinerea*, and *R. stolonifer,* by three in vitro methods followed by an in situ assessment. MPEO was the least active in all in vitro assays. It showed the highest MIC > 1000 μL/L (1000 μg/mL) (*C. cyminum* 750 μL/L = 750 μg/mL), and EC_50_, as well as the lowest percentage inhibition of the radial growth [80]. In the next step, strawberries were dipped in a conidia suspension, then dipped or sprayed in a 1000 μL/L (1000 μg/mL) EO solution, and stored for 10 days at 7 °C. In both in situ variants, MPEO appeared to be the most effective. The percentage of rot incidence caused by the three fungi was lower than 21% after spraying and <13.3% after dipping. At the same time, *C. cyminum* oil showed 22–75% and 13–74% incidence, and for synthetic fungicide tiabandazol used in a concentration of 1.5:1000 it was 33.5–62.8% and 33.5–62.5%, respectively. Pure menthol was also effective at suppressing fungal growth in strawberries and extending the shelf life, although thymol was more active. Strawberries maintained better quality, with higher levels of sugars, organic acids, phenolics, anthocyanins, and flavonoids than the untreated fruits [81].

Chaemsanit et al. [82] showed that MPEO adsorbed activated carbon at an oil concentration of 700 μL/L air inhibited surface mold of four fungi and prolonged the shelf life of dragon fruit during 14 days’ storage. Moreover, EO maintained the fruit’s firmness, greenness, acids, and phenolic content. The main oil components, menthol and menthone, showed lower antifungal activity than the oil. The authors concluded that other constituents of EO may display some synergistic effects.

MPEO was also effective in preharvest treatment to control lettuce deterioration during postharvest storage. The natural fungicide Fungastop^TM^, containing 0.2% peppermint oil and 0.4% citric acid as active substances, sprayed on lettuce before harvesting had the same efficacy as chemical fungicides in reducing microbial spoilage (total mesophilic bacteria, molds, yeast) and increasing lettuce shelf life [83]. Fungastop^TM^ was recommended as an alternative to synthetic fungicides.

The efficacy of MPEO was tested against the *Agrobacterium* bacterium that causes crown gall in orchards. MIC of MPEO for nine genomovars of *Agrobacterium tumefaciens*, *A. viridis*, and *A. rhizogenes* varied in the broad range 0.01–12.5 mg/mL (10–12500 μg/mL), while for tetracycline it was 0.05–200 μg/mL. In a pot greenhouse experiment, different EO solutions (100,000–250,000 μg/mL) were tested toward the most susceptible strain, *A. tumefaciens* ATCC 23308^T^ (MIC 10 μg/mL), on one-month-old tomato plants. EO was placed topically on wounded internodes inoculated with 10 μL of *A. tumefaciens* suspension, and stored for one month. MPEO at 200,000 μg/mL completely inhibited the formation of tumors and at lower doses decreased the weight of tumors. No phytotoxic effects were recorded on tomato plants [84].

MAEO was a potent fumigant for the management of the biodeterioration of stored wheat [77]. At a concentration of 1300 ppm (1300 μg/mL) in vapor, it efficiently controlled seven toxic fungi in the uninoculated wheat sample. After the inoculation of wheat with *A. flavus*, fungal growth was controlled for 12 months. What is more, the oil also controlled the insects *Sitophilus oryzae* and *Tribolium castaneum*.

MPEO was selected by in vitro diffusion testing as the most effective out of 32 EOs against bacterial fruit blotch of watermelons caused by *Acidovorax citrulli*. Contaminated seeds are one of the bacterial sources in fruit. MPEO and its components (menthol, neomenthol, isopulegone, and 1,8-cineole), tested in vivo in the decontamination of inoculated watermelon seeds, appeared to be effective at preventing bacterial growth after soaking seeds in a 0.2% MPEO solution [42].

Among the 11 EOs studied for maize kernel protection against *A. flavus*, peppermint oil belonged to the six most effective. Maize was immersed in EO solution, inoculated by fungi, and incubated for five days. The contaminated grains were counted. The protective dosage resulting in total *A. flavus* growth inhibition was 5% for clove and basil oil and 8% for peppermint oil. A synergistic effect was observed between cinnamon oil and other oils. No inhibition of maize germination was observed after treatment [56].

When tested in vitro against two strains of *Xanthomonas oryzae* that cause bacterial diseases in rice, MPEO was less active than citronella and eucalyptus EOs. These two EOs in effective antibacterial dilutions reduced rice seed germination and induced a herbicidal effect on rice leaves [85].

It is hardly possible to control plant diseases using just one strategy. The integrated effect of mycorrhizal fungi (*Glomus mosseae*) and EOs was investigated against the bacterial wilt disease of tomato caused by *Ralstonia solanacearum* [86]. Two the most active out of nine EOs tested in vitro (disc diffusion method), MPEO and thyme oil, as well as their combinations, were selected for greenhouse and field experiments. The roots of tomato seedlings were treated with EO at the time of transplanting and the soil was inoculated with bacteria. After four weeks, the disease index was recorded. In field conditions, thyme oil exhibited the highest disease reduction (>94%), followed by a thyme‒peppermint oil combination (>82%). *G. mosseae* exhibited low disease reduction; however, it increased the yield [86].

The antibacterial and antifungal efficacy of EO can be improved by using mixtures of EOs or their combinations with other agents. Some experiments were devoted to MPEO and chitosan mixtures. Combinations of MPEO or menthol and an adjuvant, aluminum starch octenylsuccinate, in different amounts were tested against *Fusarium sambucinum* and *Rhizoctonia solani.* Menthol was significantly more effective than MPEO (96% menthol) against 12 fungal potato storage pathogens (among them, four *Fusarium* and two *Phytophthora* species) and showed 100% inhibition in a vapor test (100 μL in a 9-cm Petri dish), while MPEO inhibited *Fusarium avenaceum* by 92.7% and *Phoma exigua* by only 3%. A mixture of adjuvant (10 g) and EO (4 and 8 g) used at 200 mg per Petri dish significantly prolonged the inhibitive activity due to the control of the release of volatiles [87].

An interesting approach to preventing bacterial and fungal decay in fresh fruit and vegetables is using natural, edible, and biodegradable polysaccharide and protein-based coatings. A natural polysaccharide, chitosan, itself possesses antimicrobial activity [88], and was used in combination with MPEO in research done by de Oliveira and Guerra’s group [89,90,91]. In research by de Oliveira et al. [89], different concentrations of MPEO, chitosan, and their combinations were shown to be effective against five *Colletotrichum* species causing anthracnose in mangoes (*C. asianum*, *C. fruticola*, *C. tropicale*, *C. dianesei*, and *C. karstii*). In the agar dilution method, total inhibition of mycelial growth was observed at 5 μL/mL of peppermint oil and at 10 mg/mL of chitosan. The same efficacy was shown for the combination of 0.6 μL/mL oil and 5 mg/L chitosan. It was proven that additive or synergistic effects occurred. In the next step, mango fruits inoculated with the tested strains were immersed in dispersions containing mixtures of MPEO (0.6 or 1.25 μL/mL) and chitosan (5 or 7.5 μL/mL) and stored at 4 °C for 30 days. The anthracnose lesion severity during the storage of mangoes coated with each of these mixtures was similar to or lower than that observed with synthetic fungicides (5 or 10 mg active ingredient/mL).

Combinations of chitosan and two mint oils, MPEO and *M. villosa* essential oil, were used as edible coatings protecting cherry tomato fruits [90] and table grapes [91] from postharvest mold induced by *A. niger*, *B. cinerea*, *P. expansum*, and *R. stolonifer.* The MIC values, assessed by macrodilution in vitro assay against all fungi, were 8 mg/mL for chitosan and 5 μL/mL for MPEO. Fruits were immersed in spore inoculum for 1 min, dried, immersed in a combination of chitosan and MPEO solutions at different concentrations, and then stored at room temperature for 12 days and at low temperature for 24 days. The coating, comprising chitosan 4 mg/mL/MPEO 1.25 and 2.5 μL/mL (1250 and 2500 μg/mL), delayed mold growth and reduced infections caused by all fungi in grapes and cherry tomatoes at room or low temperature without altering the taste of the fruit [90,91].

An alternative to using EOs for protecting food commodities is active packaging. This is when EOs are incorporated into or coated onto packaging films [92] or used as a vapor in modified atmosphere packaging, e.g., in the headspace or entrapped in sachets able to slowly release the active compounds [93]. In experiments done by a Spanish group, oxygenated monoterpenes were assessed as active agents. Menthol, thymol, carvacrol, and eugenol in the vapor phase were effective at reducing the occurrence of table grapes’ decay [94,95]. After 35 days of cold storage, the microbial population (total mesophilic bacteria, molds, yeast) was drastically reduced. Decay was reduced with no effects on the grapes’ firmness. Fruit samples treated with oil vapors did not differ in terms of percentage weight loss, organic acid content, sweetness, and total phenolic content during or following vapor exposure, compared with untreated fruit.

## 6. Insecticidal and Acaricidal Activity of Peppermint Oil and Cornmint Oil

A thorough review of the insecticidal activity of mint EOs was performed in 2011 by Kumar et al. [96]. In this review, the authors charted the development of methods for studying the insecticidal potential of mint EOs. However, they also pointed to missing elements in the research designs, such as the lack of formulations and lack of field trials [96]. Since then, some progress has been made, although studies of the insecticidal potential of MPEO and MAEO are still mostly performed in the laboratory, as summarized in Table 3.

There are three basic techniques used for the in vitro assessment of the insecticidal activity of essential oils. The first is a contact toxicity assay, in which insects are exposed to direct contact with the EO. The second is a fumigant assay, in which insects are exposed to the vapors of EOs, but without direct contact with them. The third is an acute toxicity assay, in which the EOs are mixed with the food for insects, i.e., grains or green roughage. The experiments are usually performed in Petri dishes, vials, or jars of different volumes, depending on the species of insect and the developmental stage. There are usually a few different doses of the same EO applied in one experiment, and the assessments are performed after different periods of time (from 6 h up to 72 h after the application of the EO). As a result, a dose causing 50% death in the examined insects (lethal concentration, LC_50_; lethal dose, LD_50_ or effective dose, ED_50_) or a median knockdown time (KT_50_—the time after which 50% of the tested insects are knocked down), or a dose repelling 50% of insects (RI_50_), is calculated. In the research papers discussed in this review, as for the fungicidal bioassays, there were significant discrepancies in the experimental designs, i.e., different doses of EOs, terms of measurements, and conditions of studies, e.g., different temperatures, volumes of vials, etc. Also, the EOs were applied in a variety of forms, either as pure oils, EOs dissolved in organic solvents, e.g., acetone, or in the vegetable oils, or as oil-in-water (nano)emulsions, e.g., with Tween 80 as an emulsifier. All of the factors mentioned above, as well as the differences in the chemical composition of the EOs, meant that the results of individual experiments are not comparable.

### 6.1. Against Storage Insects

MPEO, tested as a volatile in laboratory conditions (a Petri dish), displayed strong repellency and toxic effects against cowpea weevil (*Callosobruchus maculatus* F.), with a 50% lethal concentration (LC_50_) of MPEO by 25.70 μL/L of air (0.0257 μL/mL of air) [97]. MPEO activity against the Indianmeal moth (*Plodia interpunctella* Hübner) was tested with the other five EOs [98]. The contact toxicity of MPEO against adults of *P. interpunctella* after 24 h was 53.8 μg/cm^2^, which was about half that of the most active EO of palmarosa (*Cymbopogon martinii* (Roxb.) Wats). As for the fumigant knockdown effect of EOs against adults of *P. interpuctella*, the most effective was the EO of eucalyptus, whereas the MPEO was one-third as active (KT_50_ = 27.1 min) [98]. Another approach against *P. interpunctella* was the contact action of MPEO loaded within polylactic acid (PLA) nanofibers (14 wt %) [99]. The authors exposed the first larvae stage of *P. interpunctella* to MPEO loaded in PLA nanofibers for 72 h at 27 °C and 65% relative humidity. PLA, being a biodegradable polymer, effectively loaded the MPEO particles and produced a safe pesticide. It was found that the LC_50_ of the MPEO in the nanofibers was 30.3 μL/L of air. The authors concluded that the use of MPEO, with a short period of toxicity, causes nanofibers to be considered as a new formulation of pesticides in storage [99].

The fumigant potential of MPEO against *Sitophilus oryzae* (L.), the rice weevil, one of the most destructive insect pests of stored cereals worldwide, was investigated in a few studies. They returned contrasting results, clearly pointing to a significant effect of chemical composition, concentration of MPEO, and duration of action, for a final insecticidal outcome. The MPEO was significantly effective in *S. oryzae* at a 400 μL/L air concentration, inducing 83% and 100% mortalities in with-food and without-food conditions, respectively, during a 72-h exposure [100]. In addition, these authors observed that binary mixtures of peppermint oil and lemon oil (1:1 ratio) produced an equivalent effect to MPEO alone [100]. Rajkumar et al. [101] also underlined that both fumigant toxicity and the antifeedant activity of MPEO could be effective methods of *S. oryzae* control. At a LC_50_ dose of MPEO = 43.17 μL/L air (0.043 μL/mL air), the authors obtained an antifeeding deterrent effect equal to 100%. The toxicity of the MPEO, tested in this experiment, in comparison with its main compounds, namely menthone and menthol, was comparable each time [101]. To the contrary, in other laboratory research [102], an antifeeding deterrence effect (85%) of MPEO over its fumigant toxicity (ED_50_ 0.3 μL/mL air) and feeding inhibitory effect against *S. oryzae* was proven. At the same time, the fumigant toxicity and repellence activity of MPEO turned to be, respectively, about four and five times less toxic than hyssop (*Hyssopus officinalis* L.) EO [102]. Inhibition of acetylcholinesterase activity, combined with an oxidative imbalance, is given as a potential mode of action of MPEO on *S. oryzae* [101]. The inhibition of acetylcholinesterase activity, accompanied by oxidative imbalance, was also proposed as a mechanism of action of sublethal LC_50_ doses of MPEO and MAEO against *Tribolium castaneum*, a pest of stored grains [101,103].

### 6.2. Against Herbivory Insects

MPEO showed a satisfactory level of control of melon aphids (*Aphis gossypii* Glover), in a Petri dish experiment, when used alone as vapors or combined with the application of the pathogenic fungus *Lecanicillium muscarium* (Zare and Gams) [104]. The LC_50_ for MPEO vapors was 15.25 μL/L of air. Aphid mortality increased when essential oils of MP were combined with *L. muscarium*; however, the phenomenon was additive rather than synergistic. At the same time, the mycelial growth inhibition of *L. muscarium* exposed to the MPEO was low, which allowed for the combined use of both [104].

The repellent action of MPEO against apterous females of the bird cherry-oat aphid (*Rhopalosiphum padi* L.) was verified in a laboratory experiment, along with the other eight EOs as well as their main compounds. MPEO at a dose of 0.15 μL/cm^2^ was one of the three most repellent EOs, along with aniseed and lemongrass EOs, with a repellency index R.I. = 72 in an airtight box. A repellent dose of the ultrasound-assisted water nanoemulsion with MPEO (5%) and Tween 80 (1:1), sprayed precisely at the surface of a barley leaf at a dose of 0.13 μL/cm^2^, resulted in R.I. = 58.4 after 24 h [105].

MPEO was effective at killing third-instar nymphs and adults of mealybugs (*Planococcus ficus*) feeding on grapevine (*Vitis vinifera* L.). The reference product was paraffin oil. As a result, 24 h after treatment with pure MPEO, a significant number of dead insects was noted, with LD_50_ of MPEO equal to 8.1 mg/mL (8100 μg/mL) against adult insects and 5.4 mg/mL (5400 μg/mL) against third-instar nymphs. MPEO was about 25–50% more toxic compared to the paraffin oil. At the same time, the authors performed a phytotoxic study of MPEO, which showed some negative effects on grapevine leaves in the form of necrotic spots; however, the leaf surface percentage with symptoms did not exceed 25%, at a dose > 9 mg/mL (9000 μg/mL) [11].

The sensitivity of spotted wing drosophila (*Drosophila suzukii* Matsumura) to several EOs, including MPEO, in fumigant and contact toxicity assays, was tested by Park et al. [106]. MPEO showed effective fumigant activity as compared to the other EOs tested. Mortality by fumigation with MPEO at 5.88 μg/mL air was >98% (LC_50_ = 3.87 μg/mL air) and 100% (LC_50_ = 4.10 μg/mL air) against males and females, respectively. Contact toxicity 24 h after treatment with MPEO was >90% against males, at a concentration of 20 μg/fly, but against females it was much lower. Menthone and menthol were identified as the major components of *M. piperita*. The LC_50_ (μg/mL) values of menthone and menthol were 5.76 and 1.88 against males and 5.13 and 1.94 against females, respectively [106].

MPEO was tested, along with the other six EOs, in a set of laboratory bioassays against *Plutella xylostella*, a pest of economic importance for Cruciferae [107]. As a result, a promising repellent concentration value (RC_50_ = 1330 μg/mL) of MPEO against third-instar larvae of *P. xylostella* was calculated. The repellence of the larvae at different concentrations of MPEO was 74.86% at 10,000 μg/mL and 46.29% at 1250 μg/mL. MPEO also caused inhibition of growth of third-instar larvae by 67.26% at 10,000 μg/mL, 35.26% at 500 μg/mL, and 9.87% at 2500 μg/mL. The authors concluded that the MPEO, as well as the EOs of *Mentha longifolia* (L.) and *Curcuma aromatica* (Salisb.), were most efficient against larvae of *P. xylostella*, and their combinations with other botanical formulations/extracts/oils can be studied further under field conditions [107].

The effectiveness of nine EOs, including MAEO, was tested against *Meligethes aeneus*, another important pest of Cruciferae, especially oilseed rape (*Brassica napus* L.) across Europe [108]. The study was performed in the laboratory using different types of insecticidal activity: acute and repellent. MAEO showed low toxic activity against adults of *M. aeneus*, as compared to the other EOs, with LD_50_ of 964 and 548 μg/cm^2^ after 6 h and 24 h of exposure, respectively. The most active were the EOs of *Carum carvi* (L.) and *Thymus vulgaris* (L.), with LD_50_ values of 197 and 250 μg/cm^2^, respectively. The highest repellency of 10 μL/mL of MAEO was 89.4% after 1 h, decreasing to only 5.1% after 24 h of exposure. That was much lower compared to the most active EOs of *C. carvi* and *T. vulgaris*, where significantly highest repellent indexes of 65.6% and 63.8%, respectively, were determined. At the same time, more than 50% of oilseed rape buds burst into blossom in the presence of MAEO oil, showing its protective role against *M. aeneus* [108].

### 6.3. Against Livestock Insects

Khater [109] studied the effect of water emulsions containing different concentrations of MPEO or three oils (pumpkin, lupine, and garlic) against larval stages of *Cephalopina titillator,* the parasite responsible for nasopharyngeal myiasis in camels. The positive control group was treated with ivermectin and the negative control was treated with distilled water with a few drops of Tween 80. The author applied in vitro larval immersion (dipping) tests. All the larvae of the parasite died after 24 h from the application of a 7.5% concentration of MPEO, whereas the LD_50_ was 0.42%. The most effective against the insects, however, was ivermectin (0.15%) with LD_50_ = 0.03%, followed by 2% pumpkin oil with ED_50_ = 0.2%. Formation of pupae had been stopped after treatment of larvae with 7.5% MPEO and adult emergence had ceased following treatment of larvae with 0.5% MPEO. MPEO was also the cause of several morphological abnormalities in the larvae and pupae of *C. titillator* [109].

One of the few in situ studies with mint oils was the study by Lachance and Grange [110]. They reported the repellent action of eight EOs (basil, geranium, balsam fir, lavender, lemongrass, peppermint, pine, and tea tree) applied singly with sunflower oil or ethyl alcohol, as carriers, against horn flies (*Haematobia irritans*) on pastured dairy cows and heifers. Safer’s soap, natural pyrethrins without piperonyl butoxide, and ethyl alcohol alone were used as negative controls. The authors applied the EOs, at a 5% concentration in sunflower oil, to the flanks of animals less than 30 min before the cows were released to pasture in the morning or to the heifers in the barn. The mixtures with MPEO repelled >75% of flies on the treated area for 6 h and 8 h on pastured cows and indoor heifers, respectively. Geranium, lemongrass, and peppermint EOs were effective for a longer duration. Essential oils mixed with ethyl alcohol demonstrated less repellence than when mixed with the carrier oil. The authors concluded that the EOs could be formulated for use as fly repellents in livestock production [110].

### 6.4. Acaricidal Effects

MPEO displayed a good acaricidal effect in the laboratory experiment against a store-food mite, *Tyrophagus putrescentiae*. The mite species was tested in the laboratory bioassays against the MPEO and menthol isomers, in comparison to synthetic acaricide benzyl benzoate [111]. As a result, all but one isomer of menthol, except (+)-isomenthol, were several times more effective against *T. putrescentiae*, as compared to reference benzyl benzoate and MPEO. The LD_50_ for MPEO was equal to 2.72 and 1.87 μg/cm^2^ for the fumigant and filter paper bioassays, respectively. The relative toxicities, calculated as in previous studies, were 4.3 μg/cm^2^ and 4.5 μg/cm^2^ for the fumigant and filter paper bioassays with MPEO, respectively. Those values were about four times more effective against *T. putrescentiae* as compared to benzyl benzoate [111]. MAEO caused total mortality of the mite at doses of 5–20 μg/cm^2^, with an LD_50_ value of 3.41 μg/cm^2^. The relative toxicity of MAEO, calculated as a proportion of LD_50_ of (benzyl benzoate) versus the LD_50_ of MAEO, was 3.52-fold more active than that of the acaricide [112]. The same 

## 7. Peppermint and Cornmint EOs as Candidates for Botanical Herbicides

The allelopathic potential of *Mentha piperita* has been thoroughly examined, especially for the antigerminative effects of its water extracts against different weeds and crops [113,114,115].

Also, MPEO displays significant antigerminative potential, as reflected in a few laboratory experiments. However, the final results vary depending on the dose of MPEO, but also on its chemical composition, the conditions of the experiment, and the tested species (Table 4).

An effective dose of MPEO, causing 50% inhibition of seed germination (ED_50_), was in a range of 1030 μg/mL for maize kernels to 60 μg/mL for *Centaurea cyanus* (L.) seeds. These results placed MPEO in the group of the most phytotoxic EOs, next to *Carum carvi*, *Thymus vulgaris*, and *Salvia officinalis* [116]. In another laboratory experiment, MPEO caused a total inhibition of germination of *Lolium multiflorum* at a dose of 0.125 mL/L (125 μg/mL), but at the same time did not affect the germination of *Portulaca oleracea*, *Echinochloa crus-galli*, and food crops, e.g., maize, rice, and tomatoes, at doses from 0.125 μL/mL to 1 μL/mL (125 μg/mL to 1000 μg/mL). The most effective in this study, causing a total inhibition of seed germination even at the lowest dose, was *Satureja montana* (L.) EO [117]. Interestingly, in another two experiments, the same species, *Portulaca oleracea*, was also subjected to MPEO, and the authors were able to reach a complete inhibition of its germination. However, the inhibiting doses of MPEO for this species were 1000-fold different: 1.8 mL/L (1800 μg/mL) [118] and 1.8 μg/mL [120=119]. These doses also inhibited the germination of other species in both experiments: *Convolvulus arvensis* and radish [118], and *Amaranthus retroflexus* and *Vicia sativa* [119]. Smaller doses of MPEO, from 0.25 to 1.250 mg/L (0.25–1.250 μg/mL), did not show any inhibitory effect against the germination of an invasive species in Europe, *Solidago canadensis* (L.) [120].

MPEO not only inhibits seed germination, but also slows down the germination time, i.e., in the case of tomato seeds, the germination was slowed down by the MPEO oil-in-water emulsion (1 mL/L = 1000 μg/mL) by about six days, as compared to the untreated control [121]. Moreover, MPEO significantly reduces the lengths of the coleoptile and radicle in germinating weeds, but also in crop seedlings [116,117,118].

All of those effects make MPEO a prospective future soil-applied product, and its phytotoxic potential is also being studied in the pot experiments, using different formulations. For example, in a greenhouse pot experiment MPEO was sprayed as an oil-in-water emulsion on a surface of a sterilized loamy soil, at a volume of 5 mL per 78 cm^2^. The emulsion showed 82% inhibition for the emergence of *A. retroflexus* and 62% for *Lolium* spp. at a concentration of 345.6 mg/L (345.6 μg/mL), and 44% inhibition for the emergence of *Sinapis arvensis* at a concentration of 86.4 mg/L (86.4 μg/mL). However, in this study the most effective against germination of the tested weeds was cinnamon EO [122]. In another pot experiment, a significant inhibitory effect, of about 50%, of soil-applied MPEO microencapsulated in maltodextrin (12% concentration of EO), at a dose equal to 20 g/m^2^, against biomass accumulation of *E. crus-galli* was showed. At the same time, the microencapsulated MPEO did not exhibit inhibitory action against the accumulation of biomass in the shoots of maize, but the accumulation of chlorophyll as well as the biomass of roots was reduced by about 50%, as compared to the maltodextrin alone [123].

Results on the herbicidal activity of MAEO are much scarcer. In the pot experiment, four-week-old plants of *Anagallis arvensis*, *Cyperus rotundus*, and *Cynodon dactyalon* were sprayed a few times at 15-day intervals with oil-in-water emulsions containing different concentrations of MAEO with 0.5% (*v*/*v*) Tween 80. The emulsions were effective against *A. arvensis*, but also against *C. dactyalon* at a concentration of 100 µL/mL (100,000 μg/mL) [124]. In the same experiment, the authors also proved the antimicrobial effects of MAEO against soil microbes. On the other hand, the oil increased the activity of soil enzymes, namely β-glucosidase and alkaline phosphatase [124].

## 8. Formulations of Menthol Mint Essential Oils in Agriculture—Limitations and Perspectives

Essential oils are mixtures of nonpolar volatile compounds; hydrophobic in nature, EOs do not dissolve in water and are unstable in the presence of light and oxygen. These features are major limitations on the broad application of essential oils in agriculture. The others include intensive flavor and high prices. Fortunately, these restrictions are balanced by the unquestionable advantages of EOs, i.e., biodegradability and low toxicity to human beings and other mammals. What is more, these limitations can be overcome by using different techniques for formulating the EOs.

In the previous sections, some suggestions for how to improve menthol mint EOs’ efficacy in controlling pests have been given, e.g., using EO mixtures or combinations of EOs with other agents, or using EOs in the vapor phase. Although some EOs revealed higher antimicrobial activity than MPEO and MAEO, it is worth mentioning that in mixtures of EOs additive and synergistic effects were observed [56,63,76]. Synergistic effects resulting from combinations of EOs instead of a single EO are of great significance, especially in food preservation. Essential oils can affect the sensory appeal of odor and flavor of products positively or negatively. It is important that preservative addition should not impair the quality of the product. Mixtures of EOs are more likely to be accepted by consumers than pure EO. Comparing the aroma characteristics of fruit and EOs, it was concluded that a combination of sweet and/or fresh flavors like cinnamon, clove, basil, citrus, and peppermint EOs could increase the value and acceptability of fresh-cut fruit like apples, pears, strawberries, pineapple, and some citrus fruits, both alone and in fruit salad [125].

Among the methods of improvement of EOs, efficacy encapsulation in different matrices seems to be the most novel. Maes et al. [126] recently reviewed the possibility of using this process to develop biosourced pesticides. Encapsulation protects EOs from evaporation and chemical degradation, but also enables their controlled release. Both aspects are useful in agriculture. The applicability of encapsulation to MPEO was previously researched with maltodextrin [127], modified starches [128,129], and gelatin/gum Arabic [130] as core materials. Additionally, menthol was encapsulated in cyclodextrin [131]. It was shown that encapsulation was effective for retaining of PMEO and its sustaining release [128,129,130]. The inclusion of menthol in cyclodextrin enhanced the water solubility and thermal stability of menthol [131]. What is more, it was proven that encapsulation enhanced the antimicrobial activity of EOs [132]. Although the possibility of encapsulation of mint oils has been researched, to date there are scarce data on using encapsulated menthol mint oils in crop protection. The use of encapsulated MPEO as an herbicide was mentioned in another study [123]. The encapsulation of MPEO essential oil in a chitosan‒cinnamic acid nanogel significantly enhanced its antimicrobial activity against *A. flavus.* MIC values under sealed condition were 2000 and 500 ppm (2000 and 500 μg/mL) for pure and encapsulated oil, respectively. Under unsealed conditions, the MIC of encapsulated PMEO was 800 ppm (800 μg/mL), while pure oil showed no activity at 3000 ppm (3000 μg/mL). What is more, encapsulated oil preserved tomato fruits at 1000 ppm during a one-month storage and delayed the decay process [133].

In summary, menthol mints can replace synthetic pesticides in controlling specific groups of pests, such as select species of phytopathogenic microorganisms, mites, or weeds. The varied sensitivity of various pests and crop species creates an opportunity for the selective action of these natural compounds, which is their important advantage. Although a lot of in vitro research conducted in the laboratory is available, especially on antimicrobial activity, the limitation is the narrow scope of the research performed in in situ conditions as well as a lack of knowledge on the modes of action of the menthol mint EOs, especially for insect pests and weeds.

## Figures and Tables

**Table 1:** In vitro antifungal and antibacterial activity of peppermint oil and cornmint oil against phytopathogens.

Fungi/Bacteria (B)	MIC or Total Inhibition Concentration	No. of Essential Oils Mint Oil Composition [%]	Methods Results for the Most Active Essential Oil	Ref.
*Alternaria alternata* *Alternaria solani* *Aspergillus flavus* *Aspergillus niger* *Fusarium solani* *Rhizopus solani* *Rhizopus* spp.	117.0/57.9μg/mL^1^ 127.1/129.0 122.0/110.7 49.5/63.5 130.7/89.8 44.11/63.9 149.7/137.1	4 EOs, 4 compounds MPEO menthone 28.1, menthol 4.8, menthyl acetate 9.5, limonene 7.1 MAEO, menthol 78.9, menthone 6.4	broth microdilution, agar disc diffusion (15 μL), positive control: fluconazole 30 μg *M. spicata* and *M. longifolia* similar results as MPEO	[39]
*Alternaria brassicae* *Botrytis cinerea* *Cladobotryum mycophilum* *Fusarium oxysporum* *Phytophthora parasitica* *Pythium aphanidermatum* *Sclerotinia sclerotiorum*isolated from vegetables and mushrooms	16.2%^2^ - 7.4% 15.8% 5.7% - 6%	12 EOs MPEO menthol 42.0, menthone 28.8, 1,8-cineole 7.1	disc diffusion 8 μL of 5–30% EO solution MPEO oil belonged to four most effective	[60]
*Alternaria citrii* *Aspergillus fumigatus* *Aspergillus oryzae* *Fusarium oxysporum* *Fusarium solani* *Helminthosporium compactum* *Macrophomina phaseolina* *Sclerotium rolfsii*	0.25 μL/mL 1.0 1.0 3.0 2.0 0.5 2.0 0.25	4 EOs MPEO no data	disc diffusion, 5 μL, agar dilution 0.16–20 μg/mL MPEO less active than other three	[61]
*Alternaria citrii* *Botrytis cinerea* *Colletotrichum gloeosporioides* *Lasiodiplodia theobromae* *Penicillium digitatum* isolated from fruits	3000 μL/L 3000 3000 >3000 2000	18 EOs MPEO menthol 40.7, menthone 21.7	agar dilution thyme 500–1000 μL/L (3000 *P. digitatum*)	[47]
*Aspergillus ochraceus*	2000 μg/L (broth) 1500 μg/L (vapor)	5 EOs, 5 compounds MPEO menthol 50	broth dilution/vapor phase cinnamon oil and cinnamaldehyde: 250–500 μg/L (broth), 150–250 μg/L (vapor), ochratoxin A production inhibited at 200 μg/L	[53]
*Aspergillus ochraceus*	1000 ppm	4 EOs MAEO, no data	broth dilution MAEO and oregano oil were the most effective in inhibition of fungal growth and ochratoxin A production	[52]
*Aspergillus flavus* *Aspergillus niger* *Aspergillus parasiticus* *Penicillium chrysogenum*	10000 ppm 5000 2500 1250	8 EOs and EOs combinations MPEO menthol, menthone	broth dilution, vapor phase MPEO less active than thyme (312.5–1250 ppm) and oregano oils, similar activity to cinnamon oil, more active than other four oils	[62]
*Aspergillus flavus* *Aspergillus niger* *Fusarium oxysporum* *Mucor* spp. *Penicillium digitatum*	1.13/2.25 mg/mL^3^ 1.13/2.25 1.13/2.25 1.13/2.25 2.25/4.5	MPEO	agar dilution (MIC), broth dilution (MFC), well diffusion, vapor phase	[63]
*Aspergillus flavus* *Aspergillus fumigatus* *Aspergillus niger* *Botryodiplodia theobromae* *Cladosporium cladosporioides* *Fusarium oxysporum* *Helminthosporium oryzae* *Macrophomina* sp. *Sclerotium rolfsii*	0.1 mg/mL 0.1 <0.5 0.1 0.1	18 EOs MAEO menthol 73, menthone 6.1	agar dilution, positive control: four synthetic fungicides MAEO was the most efficient of EOs and more efficient than synthetic fungicides at 0.1 mg/mL four fungi were inhibited totally, other 72–100% inhibition aflatoxin B1 production by *A. flavus* inhibited at 0.05 mg/mL	[40]
*Alternaria alternata* *Aspergillus fumigatus* *Aspergillus candidus* *Aspergillus nidulans* *Aspergillus versicolor* *Cladosporium cladosporioides* *Curvularia lunata* *Fusarium nivale* *Fusarium oxysporum* *Fusarium roseum* *Penicillium* sp. *Monilia* sp. *Trichoderma viride*	400 μg/L	18 EOs MAEO no data	agar dilution, positive control: nine synthetic fungicides MAEO was the most efficient of EOs and more efficient than all synthetic fungicides at 400 μg/L 11 fungi were inhibited totally, other two >84%	[43]
*Botrytis cinerea* *Geotrichum citri-aurantii* *Phytophthora citrophthora* *Penicillium digitatum*	no inhibition at 250 ppm	19 EOs MPEO menthol 50, menthone 30, menthyl acetate 10	radial growth on plate at different concentration, positive control: four synthetic fungicides *Chrysanthemum viscidehirtum* total inhibition at 150 ppm, synthetic fungicides at 50 ppm	[64]
*Phytophthora cinnamomi* *Pyrenochaeta lycoprsici* *Verticillium dahliae*	800 ppm 400 800	8 EOs MPEO menthol 39.0, menthone 21.0, menthofuran 19.5, 1,8-cineole 7.0	agar dilution oregano 200, 50, 50 ppm, resp.	[45]
*Dreschlera spicifera* *Fusarium oxysporum* f.sp. *ciceris Macrophomina phaseolina*	1600 ppm >1600 800	MPEO menthol 25.2, menthone 30.6	agar dilution	[65]
*Colletotrichum gloeosporioides* isolated from fruits	2.0 mg/mL	28 EOs MPEO, no data	agar microdilution, positive control: amphotericin B 5–60 μL/mL coriander leaf, two lemongrass sp. 0.25 mg/mL (lemongrass oil evaluated on passion fruit)	[66]
*Fusarium* spp. *Penicillium* spp. *Phythium* spp. isolated from corn seeds	1000 μL/L 1000 >1000	18 EOs MPEO, no data	agar dilution oregano MIC 100–200 μL/L	[67]
*Mucor sp.* *Rhizopus stolonifer* *Sclerotinia sclerotiorum*	30 μL/400 mL air	2 EOs, 4 compounds MPEO menthol 33.3, menthone 29.5, 1,8-cineole 7.0	vapor phase sweet basil and menthol 30 μL/400 mL air, menthone not active	[68]
*Rhizoctonia botaticola* *Sclerotium rolfsii*	1000 μg/mL	20 EOs MAEO, no data	agar dilution 6 EOs totally inhibited both fungi’s growth at 1000 μg/mL	[69]
*Lecanicillium fungicola* var. *fungicola*	750–1000 μL/L	11 EOs MPEO menthol 39.2, menthyl acetate 20.4, menthone 15.3	broth dilution mushroom *Agaricus bisporus* MPEO was similarly active against mushroom and its pest, savory and thyme oils showed the best selectivity index	[51]
*Aspergillus niger* *Penicillium funiculosum*	11.4 μg/mL 11.4	9 EOs MPEO linalool 41.4 linalyl ac 39.5	agar dilution *Thymus letrobotrys* 2.7 and 2.2 μg/mL	[70]
*Alternaria alternata* *Aspergillus flavus* *Aspergillus fumigatus* *Cladosporium herbarum* *Fusarium oxysporum* *Aspergillus veriscolor* *Fusarium acuminatum* *Fusarium solani* *Fusarium tabacinum* *Monilinia fructicola* *Penicilliumspp.* *Rhizoctonia solani* *Sclerotinia minor* *Sclerotinia sclerotiorum* (B) *Pseudomonas syringae* (B) *Xanthomonas campestris*	1.50 μg/mL 10.0 0.50 1.50 1.50 10.0 2.50 10.0 1.50 5.50 1.50 1.50 10.0 10.0 2.50 80.0	MPEO menthol 36.0, isomenthone 23.5, menthone 24.6, menthyl acetate 9.0, menthofuran 6.9	disc diffusion 10 μL, broth microdilution, positive control: amphotericin B MIC 1–5 μg/mL menthol, menthone MIC against *P. syringae* 2.0, 1.0 μg/mL *X. campestris* 2.0, 2.0 μg/mL	[41]
*Alternaria alternata* *Aspergillus flavus* *Aspergillus niger* *Aspergillus ochraceus* *Aspergillus terreus* *Aspergillus versicolor* *Cladosporium cladosporioides* *Fusarium tricinctum* *Penicillium funiculosum* *Penicillium ochrochloron*	1.5–3.0 μL/mL in ethanol 1.0–2.5 μL/mL in Tween	4 EOs MPEO menthol 37.4, menthone 12.7, limonene 6.9, menthofuran 6.8	agar macro- (in ethanol) and micro- (in Tween) dilution, positive control: bifonazol MIC 10–15 μL/mL thyme oil 0.125–0.5 μL/mL in ethanol, 0.05–0.25 in Tween menthol 0.25–1.5 μL/mL in ethanol, 0.05–1.0 μL/mL in Tween	[71]
*Trichoderma harzianum* *Verticillium fungicola* (B) *Pseudomonas tolaasii*	3–4 μL/mL	10 EOs, 10 compounds MPEO menthol 37.4, menthyl acetate 17.4, menthone 12.7	microdilution, macrodilution, disc diffusion, vapor phase, positive control: bifonazol and prochloraz (fungi), streptomycin + penicillin (bacteria) oregano and thyme 1.5–2.0 μL/mL	[46]
(B) *Agrobacterium tumefaciens* (B) *Erwinia carotovora*		13 EOs, 14 compounds MPEO, no data	agar diffusion, 50 μL solution MPEO moderately active at 200 mg/mL 6 EOs were effective, MPEO showed weak activity	[50]
*Aspergillus flavus* *Aspergillus parasiticus* *Fusarium solani* *Sclerotium rolfsii* (B) *Pseudomonas syringae pv. phaseolicola* (B) *Pseudomonas syringae pv. tomato* (B) *Pseudomonas syringae pv. syringae* (B) *Xanthomonas campestris pv. campestris* (B) *Xanthomonas campestris pv. phaseoli*	- - - - 0.07–0.625 mg/mL 0.156–0.312 0.156–0.312 0.312–0.625 0.625–2.5	four MPEO menthol 27.5–42.3, menthone 18.4–27.9	fungi: agar diffusion, 50 μL, weak activity bacteria: microdilution menthol 0.07–1.25 mg/mL menthone 1.25–2.5 mg/mL	[54]

^1^ MPEO/MAEO; ^2^ ED_50_ concentration of 8 μL EO solution that inhibited mycelial growth by 50%; not determined; ^3^ MIC/MFC.

**Table 2:** In situ antifungal and antibacterial activity of peppermint and cornmint oil against phytopathogens.

Fungi/Bacteria (B)	No. of essential oils Mint oil Composition [%]	Host plant Methods Results	Ref.
*Botrytis cinerea* *Penicillium expansum* isolated from apples	11 EOs MPEO menthol 38.3, menthyl acetate 29.4, MAEO menthol 85.9, menthone 3.0	apples wounded fruits inoculated with spore suspension, treated with 1% and 10% EO emulsion, stored at 4 °C for 30 days, positive control: tebuconazole 125 μg/mL both mint oils at 10% were less effective than thyme and oregano oils and more active than other seven oils	[74]
*Botrytis cinerea* *Monilinia laxa* isolated from fruits	11 EOs MPEO menthol 42.1, menthone 20.8 MAEO menthol 33.3, menthone 20.8, isomenthone 10.7	apricots, nectarines, plums wounded fruits inoculated with spore suspension, treated with 1 and 10% EO emulsion, stored at 1 °C for 28 days, positive control: tebuconazole 125 μg/mL both mint oils at 10% were less effective than thyme and oregano oils and more active than other oils	[75]
*Colletotrichum asianum* *Colletotrichum fruticola* *Colletotrichum tropicale* *Colletotrichum dianese* *Colletotrichum karstii*	MPEO and chitosan menthol 41.3, isomenthone 23.5, menthone 10.8	mango in agar dilution assay total inhibition of mycelial growth at 5 mg/mL of MPEO, 10 mg/mL of chitosan, and at a combination of 0.6 μL/mL oil and 5 mg/mL chitosan wounded mango inoculated, immersed in mixtures of MPEO (0.6 or 1.25 μL/mL) and chitosan (5 or 7.5 μL/mL), stored at 4 °C for 30 days, positive control: two synthetic fungicides 10 and 5 μg a.i./mL, respectively anthracnose lesion severity was lower than with synthetic fungicide	[89]
*Aspergillus niger* *Monilinia fructicola* *Rhizopus stolonifer*	2 EOs and combinations, 4 compounds MPEO	peach in in vitro vapor test EOs at 30 μL/400 cm^3^ inhibited two molds wounded fruits inoculated by fungi, stored at 3 mL/box EO decay reduction and prolonged storage was observed	[76]
*Penicillium italicum*	20 EOs MAEO, no data	oranges and limes in agar dilution assay MAEO was the most efficient at 100 ppm better than synthetic fungicide wounded fruit inoculated and stored in 100–500 ppm in air of three the most active in vitro EOs (MAEO, *Ocimum*, and *Zingiber*) MAEO enhanced storage life from three days in control to nine and 11 days	[78]
*Botrytis cinerea*	3 EOs and combinations MPEO menthol 34.7, menthone 27.4	table grapes fruits exposed to EOs vapor for 24 h, under atmospheric and hyperbaric condition, stored in different temperature for nine days all EOs and MPEO/rosemary oil combinations controlled gray mold	[79]
*Aspergillus niger* *Botrytis cinerea* *Penicillium expansum* *Rhizopus stolonifer*	MPEO and chitosan menthol 30.3, isomenthone 26.7, menthyl acetate 8.5	table grapes fruits immersed in spore inoculum for 1 min., dried, immersed in chitosan/EO solution at different concentrations, stored in room and low temperature chitosan 4 mg/mL/MPEO 1.25 and 2.5 μL/mL delayed the mold growth and reduced infections caused by all fungi	[91]
*Aspergillus niger* *Botrytis cinerea* *Rhizopus stolonifer*	4 EO MPEO menthol 36.2, menthone 32.4	strawberry fruits dipped in the conidia suspension, then dipped or sprayed in 1000 μL/L EO solution, stored at 7 °C for 10 days, positive control: tiabandazol 1.5:1000 MPEO was the most efficient in inhibiting rot incidence, better than tiabandazol	[80]
*Aspergillus flavus* *Aspergillus niger* *Penicillium spp.* *Rhizopus* spp.	MPEO menthol 41.6, menthone 20.9, isomenthone 9.7	dragon fruit punched fruit inoculated and stored in oil vapor at 100–1000 μL/L air at 25 °C for 21 days total inhibition of fungal growth at 700, 550, 550, 425 μL/L air, resp. menthol 650–900 μL/L air; menthone 950->1000 μL/L air	[82]
*Aspergillus niger* *Botrytis cinerea* *Penicillium expansum* *Rhizopus stolonifer*	MPEO and chitosan menthol 30.3, isomenthone 26.7, menthyl acetate 8.5	cherry tomato fruit fruits immersed in spore inoculum for 1 min, dried, immersed in chitosan/EO solution at different concentrations, stored at room and low temperature chitosan 4 mg/mL/MPEO 1.25 and 2.5 μL/mL delayed the mold growth and reduced infections caused by all fungi	[90]
total mesophilic bacteria, molds, yeast	MPEO	lettuce in field experiment lettuce sprayed before harvest with Fungastop^TM^ that contained 0.2% MPEO and 0.4% citric acid, stored at 2 °C for 28 days treatment lowered microorganisms count at harvest, reduced their increase in storage, and increased shelf life	[83]
(B) *Agrobacterium tumefaciens,* 9 genomovars (B) *Agrobacterium vitis* (B) *Agrobacterium rhizogenes*	MPEO menthol 33.6, isomenthone 33.0	tomato plants MIC in broth microdilution method 0.01–12.5 mg/mL pot experiment: wounded internodes treated with EO solution (100–250 mg/mL), inoculated with *A. tumefaciens* suspension, stored for one month MPEO at 200 mg/mL completely inhibited the formation of tumors	[84]
*Ralstonia solanacearum* isolated from tomato	9 EOs MPEO, no data	tomato seedlings growth the most active in in vitro (disc diffusion) MPEO and thyme oils were examined in greenhouse and field growth: seedling roots treated with oil solution (1:100) at transplanting, inoculum added to the soil disease reduction in greenhouse: thyme oil 83%, MPEO 61%	[86]
(B) *Acidovorax citrulli*	32 EOs, 4 compounds MPEO menthol 40.8, menthone 14.7, isomenthone 8.1	watermelon seeds in disc diffusion assay only MPEO at 20 μL and basil oil at 50 μL inhibited bacterial growth seeds inoculated, soaked in EO or compound solution (0.01–0.2%) MPEO, menthol, neomenthol, and 1,8-cineole at 0.2% were effective in decontamination of watermelon seeds	[42]
*Aspergillus flavus*	11 EOs, EOs combinations, 8 compounds MPEO, no data	maize kernels immersed in EO solution (0.1–10%), sprayed with fungal spore suspension MPEO was effective at 8% and other 5 EOs at <7%	[56]
*Aspergillus flavus* *Aspergillus fumigatus* *Aspergillus niger* *Culvularia* spp. *Penicillium oxalicum* *Mucor* spp. *Rhizopus* spp.	2 EOs MAEO no data	wheat uninoculated and inoculated (*A. flavus* spore suspension) wheat treated by EO vapors at 1300 and 600 ppm, stored 12 months at room temperature 100% protection of uninoculated and 95% of inoculated wheat orange injured fruits were inoculated (*P. italicum* spore suspension), treated by EO vapors at 1500 and 1000 ppm, and stored; decay and storage life were enhanced from three to 10 days	[77]

**Table 3:** Insecticidal and acaricidal action of peppermint oil and cornmint oil.

Target Organisms	Feeding Damage	Essential oil Composition [%]	Method Formulation	Results^1^	Ref.
Insecticidal					
*Aphis gossypii* Glover	polyphagous aphid on watermelon, cotton, and vegetables	MPEO limonene 27.3, menthol 24.7, menthone 14.0	fumigant toxicity 1.87–50 μL/L air	ED_50_ 15.25 μL/L air	[104]
*Callosobruchus maculatus* F.	store pest of leguminous seeds	MPEO menthone 28.9, menthol 28.5, pulegone 6.9	fumigant toxicity 20–45 μL/L air	KT_50_ 1.89–3.29 days	[97]
			repellency test 90–360 μL/L air	R.I.71.6–87.8%	
*Cephalopina titillator* Clark	obligate parasite of camels causing Nasopharyngeal myiasis	MPEO no data	larval immersion test 0.05–60% water emulsions	ED_50_ 2.18% after 6 h and 0.47% after 24 h	[109]
*Drosophila suzukii* Matsumura	Insect pest of stone fruits and berry crops	MPEO menthol 46.4, menthone 13.8, menthofuran 7.3	fumigant toxicity 2.94–11.76 mg/L	LC_50_ males: 3.87 mg/L LC_50_ females: 4.10 mg/L	[106]
*Haematobia irritans* L.	livestock pest, bloodsucking fly	MPEO no data	repellent test 5% EO in sunflower oil on the side of pastured cows	77% less flies on the side of pastured cows after 24 h, as compared with control	[110]
*Meligethes aeneus* (Fabricius)	insect pest of oilseed rape	MAEO menthol 42.5, menthone 21.6, isomenthone 13.5	acute toxicity 50–5000 μg/cm^2^	LD_50_ 964 μg cm^−2^ after 6 h and 548 μg/cm^2^ after 24 h	[108]
			20 μL EO in 2 mL acetone sprayed on 10 yellow buds of oilseed rape	R.I. 24.6% after 6 h and 45.6% after 24 h	
*Planococcus ficus* (Signoret)	insect pest in grape vine	MPEO menthol 34.6, isomenthone 14.6, menthyl acetate 12.4, menthone 9.7, 1,8-cineole 9.3	contact toxicity 1 mL aqueous solution with 4.5, 9, and 18 mg of EO in 9-cm Petri dish	LC_50_ against 3rd instar nymphs 5.4 mg/mL LC_50_ against female adult 8.1 mg/mL	[11]
*Plodia interpunctella* Hüb.	insect pest of diverse stored food products	MPEO isomenthone 48.0, menthone 19.2, 1,8-cineole 9.2	contact toxicity 5–180 μg/cm^2^ of filter paper in Petri dish	ED_50_ 53.8 μg/cm^2^	[98]
			fumigant toxicity 40 μL per 8.5-cm Petri dish	KT_50_ 27.1 min.	
			residual toxicity assay 0.05, 0.10 and 0.15% (*w*/*w*) to 20 g of whole grain wheat	KT_50_ 3.08–11.3 days	
*Plodia interpunctella* Hüb.	--	MPEO Not assessed	contact toxicity polylactic acid nanofibers loaded with 14% (w/w) EO	ED_50_ 30.3 μL/L of air	[99]
*Plutella xylostella* L.	insect pest of cruciferous crops	MPEO menthol 40.1, menthone 31.1	residual toxicity water solutions 0.625–5.0 mg/mL	ED_50_ 1.37 mg/mL	[107]
			repellent activity leaf disks soaked with water solutions (+0.05% tritone) containing three concentrations of EO: 2.5–10.0 mg/mL	ED_50_ 1.33 mg/mL	
			growth inhibition activity 2.5–10 mg/mL after 48 h of feeding on treated leaves	pooled mean inhibition: 37.46%	
*Rhopalosiphum padi* L.	aphid pest of temperate cereal crops	MPEO menthol 40.4, menthone 23.5, menthyl acetate 8.3	repellency EO in acetone at 0.15 μL/cm^2^ in choice bioassay	ED_50_ 0.13 μL/cm^2^	[105]
			repellency water nanoemulsions, 5% Tween with 0.02, 0.29 and 0.5 μL of the EO per cm^2^; or 1% Tween with 0.06 or 0.12 μL of the EO per cm^2^	R.I. 40 (at a dose 0.02 μL/cm^2^) to R.I. 90 (at a dose 0.5 μL/cm^2^)	
*Sitophilus oryzae* L.	insect pest of stored cereals	MPEO neoisocarvomenthol 40.6, menthone 27.6, 1,8-cineole 10.6, menthyl acetate 7.5	fumigant toxicity 20–400 μL/L air, without or with food condition	ED_50_ 47.8 μL/L without food and 45.2 μL/L with food	[100]
*Sitophilus oryzae* L.		MPEO menthol 44.0, menthone 8.3, 1,8-cineole 7.1	fumigant toxicity 74.4–428.6 μL/L air	ED_50_ 299.5 μL/L of air	[102]
			repellency test 2–16 μL/30 cm^2^	R.I. 85%	
*Sitophilus oryzae* L.		MPEO menthone 31.7, menthol 17.9, piperitone oxide 9.9	fumigant toxicity 0.05–0.2 μL/mL air	ED_50_ 43.17 μL/L of air	[101]
			antifeedant activity ED_50_ 43.17 μL/L of air	FDI 100%	
*Tribolium castaneum* Herbst	insect pest of stored grains	MPEO menthone 31.7, menthol 17.9, piperitone oxide 9.9	fumigant toxicity 0.05–0.2 μL/mL air	ED_50_ 48.68 μL/L of air	
			antifeedant activity ED_50_ 48.68 μL/L of air	FDI 100%	
		MAEO No data	fumigant toxicity sublethal concentrations 40, 60 or 80% of 24 h LC_50_ (equal to 18.482 μL of MAEO)	51% less eggs produced by the insects and 33% lower pupation at an 80% of 24 h LC_50_ dose	[103]
			contraction method 30% and 60% of 24 h LC_50_ MAEO in acetone	61.2% less damaged grain at an 60% of 24 h LC_50_ dose	
Acaricidal					
*Tyrophagus putrescentiae* Schrank	mite of stored food	MPEO menthol 55.2, menthone 19.5, isomenthone 7.3	fumigant bioassay 0.63–80.0 μg/cm^2^	ED_50_ 2.72 μg/cm^2^	[111]
			filter paper bioassay 0.32–80.0 μg/ cm^2^	ED_50_ 1.87 μg/cm^2^	
		MAEO menthol 60.0, menthone 20.0	fumigant bioassay 1.25–80.0 μg/cm^2^	ED_50_ 3.41 μg/cm^2^	[112]

^1^ ED_50_, effective dose; LD_50_, lethal dose and LC_50_, lethal concentration—a dose of EO causing death of 50% of the studied insects; KT_50_; median knockdown time—a time after which a 50% of the tested insects is knocked down; R.I.—Repellency Index; FDI—feeding deterrent index.

**Table 4:** Effective doses (ED_100_ or ED_50_) of MPEO against germination of selected weeds and crop species in laboratory experiments (Petri dish).

Weed/Crop Species	ED_100_ or ED_50_	Main Compounds of EO and Their Content (%)	Ref.
*Amaranthus retroflexus* *Avena sativa* *Avena fatua* *Brassica napus* *Bromus secalinus* *Centaurea cyanus* *Zea mays*	ED_50_: 0.21 g/L ED_50_ 0.53 ED_50_ 0.72 ED_50_ 0.63 ED_50_ 0.16 ED_50_ 0.06 ED_50_ 1.03	menthone 36.8, menthol 24.0	[116]
*Portulaca oleracea* *Lolium multiflorum* *Echinochloa crus-galli* Maize Rice Tomato	70% seeds germinated at a dose of 1 μL/mL ED_100_ 0.125 μL/mL 72% seeds germinated at a dose of 1 μL/mL 6% seeds germinated at a dose of 1 μL/mL 58% seeds germinated at a dose of 1 μL/mL 3% seeds germinated at a dose of 1 μL/mL	menthone 23.3, *iso*-menthone 16.3, menthol 48.2	[117]
Radish Tomato Jungle rice *Convolvulus arvensis* *Portulaca oleracea*	ED_100_ 1.8 mL/L ED_100_ 0.6 ED_100_ 0.9 60% seeds germinated at a dose of 1.8 mL/L ED_100_ 1.8 mL/L	menthol 35.1, menthone 17.5, menthofuran 11.7	[118]
*Amaranthus retroflexus* *Chenopodium album* *Lolium spp* *Portulaca oleracea* *Sinapis arvensis* *Solanum nigrum* *Vicia sativa*	ED_100_ 1.8 mg/L ED_100_ 5.4 ED_100_ 5.4 ED_100_ 1.8 ED_100_ 5.4 ED_100_ 5.4 ED_100_ 1.8	No data	[119]

## References

[1] Salehi B., Stojanović-Radić Z., Matejić J., Sharopov F., Antolak H., Kręgiel D., Sen S., Sharifi-Rad M., Acharya K., Sharifi-Rad R. (2018). Plants of Genus Mentha: From Farm to Food Factory. Plants.

[2] Tiwari P. (2016). Recent advances and challenges in trichome research and essential oil biosynthesis in *Mentha arvensis* L.. Ind. Crop. Prod..

[3] Pushpangadan P., Tewari S.K. (2006). Peppermint. Handbook of Herbs and Spices.

[4] Bacon F.J. (1928). The Botanical Origin of American Peppermint—*Mentha Piperita* L*. J. Am. Pharm. Assoc. (1912).

[5] Maffei M., Sacco T. (1987). Chemical and Morphometrical Comparison Between two Peppermint Notomorphs. Planta Medica.

[6] Grzeszczuk M., Jadczak D. (2009). Estimation of biological value of some species of mint (*Mentha* L.). Herba Polonica..

[7] Machiani M.A., Javanmard A., Morshedloo M.R., Maggi F. (2018). Evaluation of competition, essential oil quality and quantity of peppermint intercropped with soybean. Ind. Crop. Prod..

[8] Machiani M.A., Javanmard A., Morshedloo M.R., Maggi F. (2018). Evaluation of yield, essential oil content and compositions of peppermint (*Mentha piperita* L.) intercropped with faba bean (*Vicia faba* L.). J. Clean. Prod..

[9] Ulbrich A., Kahle H., Kramer P., Schulz M. (2018). Mentha x piperita volatiles promote Brassica oleracea-A pilot study for sustainable vegetable production. Allelopath. J..

[10] Karkanis A., Alexiou A., Katsaros C., Petropoulos S. (2019). Allelopathic Activity of Spearmint (*Mentha spicata* L.) and Peppermint (*Mentha × piperita* L.) Reduces Yield, Growth, and Photosynthetic Rate in a Succeeding Crop of Maize (*Zea mays* L.). Agron..

[11] Karamaouna F., Kimbaris A., Michaelakis Α., Papachristos D., Polissiou M., Papatsakona P., Tsora E. (2013). Insecticidal Activity of Plant Essential Oils Against the Vine Mealybug, *Planococcus ficus*. J. Insect Sci..

[12] Ram M., Kumar S. (1998). Yield and Resource Use Optimization in Late Transplanted Mint (*Mentha arvensis*) under Subtropical Conditions. J. Agron. Crop. Sci..

[13] Akram M., Uzair M., Malik N.S., Mahmood A., Sarwer N., Madni A., Asif H.M. (2011). *Mentha arvensis* Linn: A review article. J. Medic. Plants Res..

[14] Singh M., Singh A., Singh S., Tripathi R.S., Singh A.K., Patra D.D. (2010). Cowpea (*Vigna unguiculata* L. Walp.) as a green manure to improve the productivity of a menthol mint (*Mentha arvensis* L.) intercropping system. Ind. Crops Prod..

[15] Chand S., Patra N.K., Anwar M., Patra D.D. (2004). Agronomy and uses of menthol mint (*Mentha arvensis*)—Indian perspective. Proc. Indian. Natl. Sci. Acad..

[16] Costa A., Chagas J., Bertolucci S., Pinto J. (2018). Growth and production of volatiles in fertilized mint. Acta Hortic..

[17] Adamović D.S., Đalović I.G., Mrkovački N.M., Pandurevic T., Bjelic D., Tyr S. (2015). Effect of growing season upon microbial status of peppermint (*Mentha x piperita* L.) rhizosphere. Acta Fytotech. Zootech..

[18] Mousavinik S.M., Asgharipour M.R., Sardashti S. (2016). Manure and Light Intensity Affect Growth Characteristics and Essential Oil of Peppermint (*Mentha piperita* L.). J. Essent. Oil Bear. Plants.

[19] Bajeli J., Tripathi S., Kumar A., Tripathi A., Upadhyay R. (2016). Organic manures a convincing source for quality production of Japanese mint (*Mentha arvensis* L.). Ind. Crop. Prod..

[20] Verma R.K., Verma R.S., Rahman L.-U., Kalra A., Patra D.D. (2016). Integrated Nutrient Management on Biomass, Oil Yields and Essential Oil Composition of Peppermint (*Mentha piperita* L.) and Residual Fertility in a Hilly Soil. J. Essent. Oil Bear. Plants.

[21] USEPA (U.S. Environmental Protection Agency) (1996). Exemption of certain pesticide substances from federal insecticide, fungicide, and rodenticide act requirements, Washington DC, USA. http://www.nj.gov/dep/enforcement/pcp/bpo/min_risk.pdf.

[22] Isman M.B. (2019). Botanical Insecticides in the Twenty-First Century-Fulfilling Their Promise?. Annu. Rev. Èntomol..

[23] Benelli G., Pavela R., Maggi F., Petrelli R., Nicoletti M. (2017). Commentary: making green pesticides greener? The potential of plant products for nanosynthesis and pest control. J. Cluster Sci..

[24] Isenring R. (2010). Pesticides and the loss of biodiversity.

[25] Maino J.L., Binns M., Umina P. (2018). No longer a west-side story – pesticide resistance discovered in the eastern range of a major Australian crop pest, *Halotydeus destructor* (Acari: Penthaleidae). Crop. Pasture Sci..

[26] Balakrishnan A. (2015). Therapeutic uses of peppermint—a review. J. Pharm. Sci. Res..

[27] Varshney S.C. (2005). Indian mint oils. Perf. Flav..

[28] (2005). European Pharmacopoeia 5.0, 5.1–5.8. Strasbourg Council of Europe.

[29] Lawrence B.M. (1993). Progress in essential oils. Perf. Flav..

[30] Telci I., Kacar O., Bayram E., Arabacı O., Demirtaş I., Yılmaz G., Özcan I., Sönmez Ç., Göksu E. (2011). The effect of ecological conditions on yield and quality traits of selected peppermint (*Mentha piperita* L.) clones. Ind. Crop. Prod..

[31] Oroian C., Covrig I., Odagiu A., Mălinaș C., Moldovan C., Fleșeriu A. (2017). Effects of Cultivation Systems and Environmental Conditions on Peppermint (Mentha × piperita L.) Biomass Yield and Oil Content. Not. Bot. Horti Agrobot. Cluj-Napoca.

[32] Santoro M.V., Bogino P.C., Nocelli N., Cappellari L.D.R., Giordano W.F., Banchio E. (2016). Analysis of Plant Growth-Promoting Effects of Fluorescent Pseudomonas Strains Isolated from *Mentha piperita* Rhizosphere and Effects of Their Volatile Organic Compounds on Essential Oil Composition. Front. Microbiol..

[33] Shahabivand S., Padash A., Aghaee A., Nasiri Y., Rezaei P.F. (2018). Plant biostimulants (*Funneliformis mosseae* and humic substances) rather than chemical fertilizer improved biochemical responses in peppermint. Iran. J. Plant Physiol..

[34] Shaikh M.N., Kasabe U.I., Mokat D.N. (2018). Influence of Rhizosphere Fungi on Essential Oil Production and Menthol Content in *Mentha arvensis* L.. J. Essent. Oil Bear. Plants.

[35] Pandey A.K., Kumar P., Singh P., Tripathi N.N., Bajpai V.K. (2017). Essential Oils: Sources of Antimicrobials and Food Preservatives. Front. Microbiol..

[36] Sarkic A., Stappen I. (2018). Essential Oils and Their Single Compounds in Cosmetics—A Critical Review. Cosmet..

[37] Kalemba D., Kunicka A. (2003). Antibacterial and antifungal properties of essential oils. Curr. Med. Chem..

[38] Van de Vel E., Sampers I., Raes K. (2019). A review on influencing factors on the minimum inhibitory concentration of essential oils. Crit. Rev. Food Sci. Nutrit..

[39] Hussain A., Anwar F., Nigam P.S., Ashraf M., Gilani A.H. (2010). Seasonal variation in content, chemical composition and antimicrobial and cytotoxic activities of essential oils from four *Mentha* species. J. Sci. Food Agric..

[40] Kumar R., Dubey N.K., Tiwari O.P., Tripathi Y.B., Sinha K.K. (2007). Evaluation of some essential oils as botanical fungitoxicants for the protection of stored food commodities from fungal infestation. J. Sci. Food Agric..

[41] Reddy D.N., Al-Rajab A.J., Sharma M., Moses M.M., Reddy G.R., Albratty M. (2017). Chemical constituents, in vitro antibacterial and antifungal activity of *Mentha piperita* L. (peppermint) essential oils. J. King Saud Univ. Sci..

[42] Choi O., Cho S.K., Kim J., Park C.G., Kim J. (2016). Antibacterial properties and major bioactive components of *Mentha piperita* essential oils against bacterial fruit blotch of watermelon. Arch. Phytopathol. Plant Prot..

[43] Kumar A., Shukla R., Singh P., Singh A.K., Dubey N.K. (2009). Use of essential oil from *Mentha arvensis* L. to control storage moulds and insects in stored chickpea. J. Sci. Food Agric..

[44] Lis-Balchin M., Deans S.G., Eaglesham E. (1998). Relationship between bioactivity and chemical composition of commercial essential oils. Flavour Fragr. J..

[45] Giamperi L., Fraternale D., Ricci D. (2002). The In Vitro Action of Essential Oils on Different Organisms. J. Essent. Oil Res..

[46] Sokovic M., Van Griensven L.J. (2006). Antimicrobial activity of essential oils and their components against the three major pathogens of the cultivated button mushroom, *Agaricus bisporus*. Eur. J. Plant Pathol..

[47] Combrinck S., Regnier T., Kamatou G. (2011). In vitro activity of eighteen essential oils and some major components against common postharvest fungal pathogens of fruit. Ind. Crop. Prod..

[48] Chao S.C., Young D.G., Oberg C.J. (2000). Screening for Inhibitory Activity of Essential Oils on Selected Bacteria, Fungi and Viruses. J. Essent. Oil Res..

[49] Baruah P., Sharma R.K., Singh R.S., Ghosh A.C. (1996). Fungicidal Activity of Some Naturally Occurring Essential Oils Against Fusarium moniliforme. J. Essent. Oil Res..

[50] El-Zemity S.R., Radwan M.A., El-Monam Mohamed S.A., Sherby S.M. (2008). Antibacterial screening of some essential oils, monoterpenoids and novel N-methyl carbamates based on monoterpenoids against *Agrobacterium tumefaciens* and *Erwinia carotovora*. Arch. Phytopathol. Plant Prot..

[51] Mehrparvar M., Goltapeh E.M., Safaie N., Ashkani S., Hedesh R.M. (2016). Antifungal activity of essential oils against mycelial growth of *Lecanicillium fungicola* var. fungicola and Agaricus bisporus. Ind. Crop. Prod..

[52] Basílico M.Z. (1999). Inhibitory effects of some spice essential oils on *Aspergillus ochraceus* NRRL 3174 growth and ochratoxin A production. Lett. Appl. Microbiol..

[53] Hua H., Xing F., Selvaraj J.N., Wang Y., Zhao Y., Zhou L., Liu X., Liu Y. (2014). Inhibitory Effect of Essential Oils on *Aspergillus ochraceus* Growth and Ochratoxin A Production. PLOS ONE.

[54] Işcan G., Kïrïmer N., Kürkcüoǧlu M., Başer H.C., Demïrcï F. (2002). Antimicrobial Screening of *Mentha piperita* Essential Oils. J. Agric. Food Chem..

[55] Ahmad A., Darokar M.P., Tandon S. (2011). Antimicrobial activity of isolates and derivatives of Indian *Mentha arvensis* essential oil. J. Essent. Oil Bear. Plants.

[56] Montes-Belmont R., Carvajal M. (1998). Control of Aspergillus flavus in maize with plant essential oils and their components. J. Food Prot..

[57] Tsao R., Zhou T. (2000). Antifungal activity of monoterpenoids against postharvesr pathogens *Botrytis cinerea* and *Monilinia fructicola*. J. Essent. Oil Res..

[58] Abbaszadeh S., Sharifzadeh A., Shokri H., Khosravi A.R., Abbaszadeh A. (2014). Antifungal efficacy of thymol, carvacrol, eugenol and menthol as alternative agents to control the growth of food-relevant fungi. Journal de Mycologie Médicale.

[59] Kotan R., Kordali S., Cakir A. (2007). Screening of antibacterial activities of twenty-one oxygenated monoterpenes. Zeitschrift für Naturforschung C.

[60] Diánez F., Santos M., Parra C., Navarro M., Blanco R., Gea F. (2018). Screening of antifungal activity of 12 essential oils against eight pathogenic fungi of vegetables and mushroom. Lett. Appl. Microbiol..

[61] Pattnaik S., Subramanyam V.R., Kole C. (1996). Antibacterial and antifungal activity of ten essential oils in vitro. Microbios.

[62] Hossain F., Follett P., Vu K.D., Harich M., Salmieri S., Lacroix M. (2016). Evidence for synergistic activity of plant-derived essential oils against fungal pathogens of food. Food Microbiol..

[63] Tyagi A.K., Malik A. (2011). Antimicrobial potential and chemical composition of *Mentha piperita* oil in liquid and vapour phase against food spoiling microorganisms. Food Control..

[64] Bouchra C., Mohamed A., Hassani Mina I., Hmamouchi M. (2003). Antifungal activity of essential oils from several medicinal plants against four postharvest citrus pathogens. Phytopathol. Mediterr..

[65] Moghaddam M., Pourbaige M., Tabar H.K., Farhadi N., Hosseini S.M.A. (2013). Composition and Antifungal Activity of Peppermint (*Mentha piperita*) Essential Oil from Iran. J. Essent. Oil Bear. Plants.

[66] Anaruma N.D., Schmidt F.L., Duarte M.C.T., Figueira G.M., Delarmelina C., Benato E.A., Sartoratto A. (2010). Control of *Colletotrichum gloeosporioides* (Penz.) Sacc. In yellow passion fruit using *Cymbopogon citratus* essential oil. Braz. J. Microbiol..

[67] Christian E.J., Goggi A.S. (2008). Aromatic Plant Oils as Fungicide for Organic Corn Production. Crop. Sci..

[68] Edris A.E., Farrag E.S. (2003). Antifungal activity of peppermint and sweet basil essential oils and their major aroma constituents on some plant pathogenic fungi from the vapor phase. Food/Nahrung.

[69] Sharma P.K., Raina A.P., Dureja P. (2009). Evaluation of the antifungal and phytotoxic effects of various essential oils against *Sclerotium rolfsii* (Sacc) and *Rhizoctonia bataticola* (Taub). Arch. Phytopathol. Plant Prot..

[70] El Asbahani A., Jilale A., Voisin S.N., Aït Addi E.H., Casabianca H., El Mousadik A., Hartmann D.J., Renaud F.N. (2015). Chemical composition and antimicrobial activity of nine essential oils obtained by steam distillation of plants from the Souss-Massa Re Region (Morocco). J. Essent. Oil Res..

[71] Soković M.D., Vukojevic J., Marin P.D., Brkić D.D., Vajs V., Van Griensven L.J.L.D. (2009). Chemical Composition of Essential Oils of *Thymus* and *Mentha* Species and Their Antifungal Activities. Molecules.

[72] Vieira-Brock P.L., Vaughan B.M., Vollmer D.L. (2017). Comparison of antimicrobial activities of natural essential oils and synthetic fragrances against selected environmental pathogens. Biochim. Open.

[73] Antunes M.D.C., Cavaco A.M. (2010). The use of essential oils for postharvest decay control. A review. Flavour Fragr. J..

[74] Lopez-Reyes J.G., Spadaro D., Prelle A., Garibaldi A., Gullino M.L. (2010). Efficacy of plant essential oils on postharvest control of rot caused by fungi on four cultivars of apples *in vivo*. Flavour Fragr J..

[75] Lopez-Reyes J.G., Spadaro D., Prelle A., Garibaldi A., Gullino M.L. (2013). Efficacy of Plant Essential Oils on Postharvest Control of Rots Caused by Fungi on Different Stone Fruits In Vivo. J. Food Prot..

[76] Ziedan E.H.E., Farrag E.S. (2008). Fumigation of peach fruits with essential oils to control postharvest decay. Res. J. Agric. Biol. Sci..

[77] Varma J. (2001). Efficacy of essential oils of *Caesulia axillaris* and Mentha arvensis against some storage pests causing biodeterioration of food commodities. Int. J. Food Microbiol..

[78] Tripathi P., Dubey N., Banerji R., Chansouria J. (2004). Evaluation of some essential oils as botanical fungitoxicants in management of post-harvest rotting of citrus fruits. World J. Microbiol. Biotechnol..

[79] Servili A., Feliziani E., Romanazzi G. (2017). Exposure to volatiles of essential oils alone or under hypobaric treatment to control postharvest gray mold of table grapes. Postharvest Boil. Technol..

[80] Hadian J., Ghasemnezhad M., Ranjbar H., Frazane M., Ghorbanpour M. (2008). Antifungal potency of some essential oils in control of postharvest decay of strawberry caused by *Botrytis cinerea, Rhizopus stolonifer* and *Aspergillus niger*. J. Essent. Oil Bear. Plants.

[81] Wang C.Y., Wang S.Y., Yin J.-J., Parry J., Yu L.L. (2007). Enhancing Antioxidant, Antiproliferation, and Free Radical Scavenging Activities in Strawberries with Essential Oils. J. Agric. Food Chem..

[82] Chaemsanit S., Matan N., Matan N. (2018). Effect of peppermint oil on the shelf-life of dragon fruit during storage. Food Control..

[83] Martínez-Romero D., Serrano M., Bailén G., Guillen F., Zapata P.J., Valverde J.M., Castillo S., Fuentes M., Valero D. (2008). The use of a natural fungicide as an alternative to preharvest synthetic fungicide treatments to control lettuce deterioration during postharvest storage. Postharvest Boil. Technol..

[84] Ben Hsouna A., Touj N., Hammami I., Dridi K., Al-Ayed A.S., Hamdi N. (2019). Chemical Composition and in vivo Efficacy of the Essential Oil of *Mentha piperita* L. in the Suppression of Crown Gall Disease on Tomato Plants. J. Oleo Sci..

[85] Wonni I., Ouedraogo S.L., Ouedraogo I., Sanogo L. (2016). Antibacterial activity of extracts of three aromatic plants from Burkina Faso againstrice pathogen, *Xanthomanas oryzae*. Afr. J. Microbiol. Res..

[86] Abo-Elyousr K.A., Seleim M.A., Abd-El-Moneem K.M., Saead F.A. (2014). Integrated effect of *Glomus mosseae* and selected plant oils on the control of bacterial wilt disease of tomato. Crop. Prot..

[87] Al-Mughrabi K.I., Coleman W.K., Vikram A., Poirier R., Jayasurija K.E. (2013). Effectiveness of essential oils and their combinations with aluminium starch octenylsuccinate on potato storage pathogens. J. Essent. Oil Bear. Plants.

[88] Devlieghere F., Vermeulen A., Debevere J. (2004). Chitosan: antimicrobial activity, interactions with food components and applicability as a coating on fruit and vegetables. Food Microbiol..

[89] De Oliveira K., Árabe R., Berger L.R.R., De Araújo S.A., Câmara M.P.S., De Souza E.L. (2017). Synergistic mixtures of chitosan and *Mentha piperita* L. essential oil to inhibit *Colletotrichum* species and anthracnose development in mango cultivar Tommy Atkins. Food Microbiol..

[90] Guerra I.C.D., De Oliveira P.D.L., Pontes A.L.D.S., Lúcio A.S.S.C., Tavares J.F., Barbosa-Filho J.M., Madruga M.S., De Souza E.L. (2015). Coatings comprising chitosan and *Mentha piperita* L. or *Mentha×villosa* Huds essential oils to prevent common postharvest mold infections and maintain the quality of cherry tomato fruit. Int. J. Food Microbiol..

[91] Guerra I.C.D., De Oliveira P.D.L., Santos M.M.F., Lúcio A.S.S.C., Tavares J.F., Barbosa-Filho J.M., Madruga M.S., De Souza E.L. (2016). The effects of composite coatings containing chitosan and *Mentha piperita* L. or *x villosa* (Huds) essential oil on postharvest mold occurrence and quality of table grape cv. Isabella. Innov. Food Sci. Emerg. Technol..

[92] Kuorwel K.K., Cran M.J., Sonneveld K., Miltz J., Bigger S.W. (2011). Essential Oils and Their Principal Constituents as Antimicrobial Agents for Synthetic Packaging Films. J. Food Sci..

[93] Serrano M., Martinez-Romero D., Guillen F., Valverde J.M., Zapata P.J., Salvador Castillo S., Valero D. (2008). The addition of essential oils to MAP as a tool to maintain the overall quality of fruits. Trends Food Sci. Technol..

[94] Valverde J.M., Guillen F., Martínez-Romero D., Castillo S., Serrano M., Valero D. (2005). Improvement of Table Grapes Quality and Safety by the Combination of Modified Atmosphere Packaging (MAP) and Eugenol, Menthol, or Thymol. J. Agric. Food Chem..

[95] Martinez-Romero D., Castillo S., Valverde J.M., Guillen F., Valero D., Serrano M. (2005). The use of a natural aromatic essential oils helps to maintain post-harvest quality of “Crimson” table grapes. Acta Horticulturae.

[96] Kumar P., Mishra S., Malik A., Satya S. (2011). Insecticidal properties of *Mentha* species: A review. Ind. Crop. Prod..

[97] Saeidi K., Mirfakhraie S. (2017). Chemical composition and insecticidal activity *Mentha piperita* L. essential oil against the cowpea seed beetle *Callosobruchus maculatus* F. (Coleoptera: Bruchidae). J. Èntomol. Acarol. Res..

[98] Jesser E.N., Werdin-González J.O., Murray A.P., Ferrero A.A. (2017). Efficacy of essential oils to control the Indian meal moth, *Plodia interpunctella* (Hübner) (Lepidoptera: Pyralidae). J. Asia-Pacific Èntomol..

[99] Somaye A., Khalil T.J., Sohrab I., Mohammad K. (2017). Contact toxicity of ploy lactic acid nanofibers loaded with two essential oils against *Plodia interpunctella* Hub. (Lepidoptera: Pyralidae). J. Biopest..

[100] Vendan S.E., Manivannan S., Sunny A.M., Murugesan R. (2017). Phytochemical residue profiles in rice grains fumigated with essential oils for the control of rice weevil. PLOS ONE.

[101] Rajkumar V., Gunasekaran C., Christy I.K., Dharmaraj J., Chinnaraj P., Paul C.A. (2019). Toxicity, antifeedant and biochemical efficacy of *Mentha piperita* L. essential oil and their major constituents against stored grain pest. Pestic. Biochem. Physiol..

[102] Khani M., Marouf A., Amini S., Yazdani D., Farashiani M.E., Ahvazi M., Khalighi-Sigaroodi F., Hosseini-Gharalari A. (2017). Efficacy of Three Herbal Essential Oils Against Rice Weevil, *Sitophilus oryzae* (Coleoptera: Curculionidae). J. Essent. Oil Bear. Plants.

[103] Mishra B.B., Tripathi S., Tripathi C. (2014). Sub-lethal Activity of Plant Volatile Essential Oils in Management of Red Flour Beetle *Tribolium castaneum* (Coleoptera: Tenebrionidae). J. Essent. Oil Bear. Plants.

[104] Ebadollahi A., Davari M., Razmjou J., Naseri B. (2017). Separate and Combined Effects of *Mentha piperata* and *Mentha pulegium* Essential Oils and a Pathogenic Fungus *Lecanicillium muscarium* Against *Aphis gossypii* (Hemiptera: Aphididae). J. Econ. Èntomol..

[105] Pascual-Villalobos M., Cantó-Tejero M., Vallejo R., Guirao P., Rodríguez-Rojo S., Cocero M. (2017). Use of nanoemulsions of plant essential oils as aphid repellents. Ind. Crop. Prod..

[106] Park C.G., Jang M., Yoon K.A., Kim J. (2016). Insecticidal and acetylcholinesterase inhibitory activities of Lamiaceae plant essential oils and their major components against *Drosophila suzukii* (Diptera: Drosophilidae). Ind. Crop. Prod..

[107] Koundal R., Dolma S.K., Chand G., Agnihotri V.K., Reddy S.G.E. (2018). Chemical composition and insecticidal properties of essential oils against diamondback moth (*Plutella xylostella* L.). Toxin Rev..

[108] Pavela R. (2011). Insecticidal and repellent activity of selected essential oils against of the pollen beetle, *Meligethes aeneus* (Fabricius) adults. Ind. Crop. Prod..

[109] Khater H.F., Ramadan M.Y., Mageid A.D.A. (2013). In vitro control of the camel nasal botfly, *Cephalopina titillator*, with doramectin, lavender, camphor, and onion oils. Parasitol. Res..

[110] Lachance S., Grange G. (2014). Repellent effectiveness of seven plant essential oils, sunflower oil and natural insecticides against horn flies on pastured dairy cows and heifers. Med. Veter. Entomol..

[111] Park J.-H., Yang J.-Y., Lee H.-S. (2014). Acaricidal Activity of Constituents Derived from Peppermint Oil against *Tyrophagus putrescentiae*. J. Food Prot..

[112] Jeon Y.-J., Lee H.-S. (2016). Chemical Composition and Acaricidal Activities of Essential Oils of *Litsea cubeba* Fruits and *Mentha arvensis* Leaves Against House Dust and Stored Food Mites. J. Essent. Oil Bear. Plants.

[113] Mahdavikia F., Saharkhiz M.J. (2016). Secondary metabolites of peppermint change the morphophysiological and biochemical characteristics of tomato. Biocatal. Agric. Biotechnol..

[114] Mahdavikia F., Saharkhiz M.J., Karami A. (2017). Defensive response of radish seedlings to the oxidative stress arising from phenolic compounds in the extract of peppermint (*Mentha × piperita* L.). Sci. Hortic..

[115] Możdżeń K., Barabasz-Krasny B., Stachurska-Swakoń A., Zandi P., Puła J. (2019). Effect of Aqueous Extracts of Peppermint (*Mentha × piperita* L.) on the Germination and the Growth of Selected Vegetable and Cereal Seeds. Notulae Botanicae Horti Agrobotanici Cluj-Napoca.

[116] Synowiec A., Kalemba D., Drozdek E., Bocianowski J. (2017). Phytotoxic potential of essential oils from temperate climate plants against the germination of selected weeds and crops. J. Pest Sci..

[117] Ibáñez M.D., Blázquez M.A. (2018). Phytotoxicity of Essential Oils on Selected Weeds: Potential Hazard on Food Crops. Plants.

[118] Mahdavikia F., Saharkhiz M.J. (2015). Phytotoxic activity of essential oil and water extract of peppermint (*Mentha×piperita* L. CV. Mitcham). J. Appl. Res. Med. Aromat. Plants.

[119] Cavalieri A., Caporali F. (2010). Effects of essential oils of cinnamon, lavender and peppermint on germination of Mediterranean weeds. Allelop. J.

[120] Jurová J., Matoušková M., Wajs-Bonikowska A., Kalemba D., Renčo M., Sedlák V., Gogaľová Z., Poráčová J., Šalamún P., Gruľová D. (2019). Potential Phytotoxic Effect of Essential Oil of Non-Native Species *Impatiens parviflora* DC. Plants.

[121] Rolli E., Marieschi M., Maietti S., Sacchetti G., Bruni R. (2014). Comparative phytotoxicity of 25 essential oils on pre- and post-emergence development of *Solanum lycopersicum* L.: A multivariate approach. Ind. Crop. Prod..

[122] Campiglia E., Mancinelli R., Cavalieri A., Caporali F. (2007). Use of essential oils of cinnamon (*Cinnamomum zeylanicum* L.), lavender (*Lavandula* spp.) and peppermint (*Mentha x piperita* L.) for weed control. Ital. J. Agron..

[123] Synowiec A., Smęda A., Adamiec J., Kalemba D., Agnieszka S., Aleksandra S., Janusz A., Danuta K. (2016). The effect of microencapsulated essential oils on the initial growth of maize (*Zea mays*) and common weeds (*Echinochloa crus-galli* and *Chenopodium album*). Prog. Plant Prot..

[124] Khare P., Srivastava S., Nigam N. (2019). Impact of essential oils of *E. citriodora, O. basilicum* and *M. arvensis* on three different weeds and soil microbial activities. Environ. Technol. Innovat..

[125] Ayala-Zavala J.F., González-Aguilar G.A., Del-Toro-Sánchez L. (2009). Enhancing Safety and Aroma Appealing of Fresh-Cut Fruits and Vegetables Using the Antimicrobial and Aromatic Power of Essential Oils. J. Food Sci..

[126] Maes C., Bouquillon S., Fauconnier M.-L. (2019). Maes Encapsulation of Essential Oils for the Development of Biosourced Pesticides with Controlled Release: A Review. Molecules.

[127] Adamiec J., Kalemba D. (2006). Analysis of microencapsulation ability of essential oils during spray drying. Drying Technol..

[128] Baranauskienė R., Bylaitė E., Žukauskaitė J., Venskutonis R.P. (2007). Flavor Retention of Peppermint (*Mentha piperita* L.) Essential Oil Spray-Dried in Modified Starches during Encapsulation and Storage. J. Agric. Food Chem..

[129] Liu C., Li M., Ji N., Liu J., Xiong L., Sun Q. (2017). Morphology and Characteristics of Starch Nanoparticles Self-Assembled via a Rapid Ultrasonication Method for Peppermint Oil Encapsulation. J. Agric. Food Chem..

[130] Dong Z., Ma Y., Hayat K., Jia C., Xia S., Zhang X. (2011). Morphology and release profile of microcapsules encapsulating peppermint oil by complex coacervation. J. Food Eng..

[131] Yildiz Z.I., Celebioglu A., Kilic M.E., Durgun T.U. (2018). Menthol/cyclodextrin inclusion complex nanofibers: enhanced water-solubility and high-temperature stability of menthol. J. Food Eng..

[132] Donsi’ F., Annunziata M., Sessa M., Ferrari G. (2011). Nanoencapsulation of essential oils to enhance their antimicrobial activity in foods. LWT.

[133] Beyki M., Zhaveh S., Khalili S.T., Rahmani-Cherati T., Abollahi A., Bayat M., Tabatabaei M., Mohsenifar A. (2014). Encapsulation of *Mentha piperita* essential oils in chitosan–cinnamic acid nanogel with enhanced antimicrobial activity against *Aspergillus flavus*. Ind. Crop. Prod..

